# Modulation of Metabolome and Bacterial Community in Whole Crop Corn Silage by Inoculating Homofermentative *Lactobacillus plantarum* and Heterofermentative *Lactobacillus buchneri*

**DOI:** 10.3389/fmicb.2018.03299

**Published:** 2019-01-23

**Authors:** Dongmei Xu, Wurong Ding, Wencan Ke, Fuhou Li, Ping Zhang, Xusheng Guo

**Affiliations:** ^1^State Key Laboratory of Grassland and Agro-ecosystems, School of Life Sciences, Lanzhou University, Lanzhou, China; ^2^Probiotics and Biological Feed Research Center, Lanzhou University, Lanzhou, China; ^3^Stay Key Laboratory of Grassland Agro-ecosystems, College of Pastoral Agriculture Science and Technology, Lanzhou University, Lanzhou, China

**Keywords:** corn silage, metabolomics, bacterial community, GC-TOF/MS, SMRT, *Lactobacillus plantarum*, *Lactobacillus buchneri*

## Abstract

The present study investigated the species level based microbial community and metabolome in corn silage inoculated with or without homofermentative *Lactobacillus plantarum* and heterofermentative *Lactobacillus buchneri* using the PacBio SMRT Sequencing and time-of-flight mass spectrometry (GC-TOF/MS). Chopped whole crop corn was treated with (1) deionized water (control), (2) *Lactobacillus plantarum*, or (3) *Lactobacillus buchneri*. The chopped whole crop corn was ensiled in vacuum-sealed polyethylene bags containing 300 g of fresh forge for 90 days, with three replicates for each treatment. The results showed that a total of 979 substances were detected, and 316 different metabolites were identified. Some metabolites with antimicrobial activity were detected in whole crop corn silage, such as catechol, 3-phenyllactic acid, 4-hydroxybenzoic acid, azelaic acid, 3,4-dihydroxybenzoic acid and 4-hydroxycinnamic acid. Catechol, pyrogallol and ferulic acid with antioxidant property, 4-hydroxybutyrate with nervine activity, and linoleic acid with cholesterol lowering effects, were detected in present study. In addition, a flavoring agent of myristic acid and a depression mitigation substance of phenylethylamine were also found in this study. Samples treated with inoculants presented more biofunctional metabolites of organic acids, amino acids and phenolic acids than untreated samples. The *Lactobacillus* species covered over 98% after ensiling, and were mainly comprised by the *L. acetotolerans, L. silagei, L. parafarraginis, L. buchneri* and *L. odoratitofui*. As compared to the control silage, inoculation of *L. plantarum* increased the relative abundances of *L. acetotolerans, L. buchneri* and *L. parafarraginis*, and a considerable decline in the proportion of *L. silagei* was observed; whereas an obvious decrease in *L. acetotolerans* and increases in *L. odoratitofui* and *L. farciminis* were observed in the *L. buchneri* inoculated silage. Therefore, inoculation of *L. plantarum* and *L. buchneri* regulated the microbial composition and metabolome of the corn silage with different behaviors. The present results indicated that profiling of silage microbiome and metabolome might improve our current understanding of the biological process underlying silage formation.

## Introduction

Ensiling is a conventional and global practice method for preserving green forage crops under anaerobic conditions. The goal of making silage is to obtain a high-quality feed for livestock. The high-quality silage should be avoided of undesirable compounds that could negatively affect animal performance, the environment, or net farm income (Kung et al., 2018). The epiphytic microbial communities of fresh forages play a critical role in the start and whole process of forage fermentation. Microbial fermentation in the silo produces an array of metabolites and can change many nutritive aspects of forage (Kung et al., 2018). However, the process of fermentation is exceedingly complex and involves many types of microorganisms, resulting in a variety of metabolites. Therefore, improved understanding of the metabolome and bacterial community in ensiled forages may provide an important scientific basis for making high-quality silages.

In the past decade, molecular tools such as denaturing gradient gel electrophoresis (DGGE), real-time PCR, terminal restriction fragment length polymorphism (TRFLP), ribosomal intergenic spacer analysis (RISA), and next generation sequencing (NGS) have been used to uncover the epiphytic microbiota in fresh forages and microbiota in ensiled forages (Stevenson et al., 2006; Brusetti et al., 2011; Li and Nishino, 2011a; Pang et al., 2011; Ni et al., 2017). However, these techniques only reflected a few of the most abundant operational taxonomic units (OTUs) present and did not reveal detailed information regarding the composition of the complete microbial community (Guo et al., 2018). Even though NGS technologies have high-throughputs and can facilitate the discovery of higher microbiota diversity as well as the detection of organisms that are presented in low numbers, partial sequence of the 16S rRNA gene analysis only provides microbiota profiles that are restricted to genus precision (Bokulich and Mills, 2012; Mayo et al., 2014). A vital metagenomic approach, the PacBio single molecule in conjunction with real-time sequencing technology (SMRT), can almost cover the full read length of the DNA fragment multiple times, resulting in a reduced error rate and increased ability to depict the bacterial profile to species level precision (Schloss et al., 2016). Hence, the SMRT sequencing platform should be considered suitable for precisely assessing the microbial community at the species level in ensiled forages with a low microbial biodiversity (Bao et al., 2016; Guo et al., 2018).

The fermentation of forage is very complex and involves many types of microorganisms mainly comprised by lactic acid bacteria (LAB), resulting in a variety of different metabolites during ensiling. Common fermentation end products of silages, such as lactic acid, acetic acid, propionic acid, butyric acid, 1,2-propanediol and ethanol, are conventionally detected to evaluate the fermentation quality of ensiled forages. Among them, acetic acid, 1,2-propanediol and propionic acid are good for improving silage aerobic stability after aerobic exposure. Some other metabolites with antifungal ability, such as 3-hydroxydecanoic acid, 3-(R)-hydroxytetradecanoic acid, 4-hydroxybenzoic acid, vanillic acid, 2,3-butanedione, acetaldehyde, and bacteriocins were also reported to be produced by LAB strains or liquid cultures of grass silage (Cleveland et al., 2001; Sjögren et al., 2003; Broberg et al., 2007; Arasu et al., 2013). In addition, LAB can produce a large number of metabolites during fermentation, such as oligosaccharides, amino acids, fatty acids, vitamins, and aromatic compounds (Sun et al., 2012). These results indicate that many other metabolites in ensiled forage might have not been identified.

To produce high quality silage, the LAB inoculants are usually used to promote fermentation process. Based on the fermentation pattern, inoculants are divided into homofermentative and heterofermentative cultures. These two types of inoculants use different approaches for directing fermentation during ensiling. Homofermentative LAB is often used to dominate the fermentation by rapid production of lactic acid and the consequent decrease in pH, which prevents growth of mold, yeast and other undesirable microbes, and helps to preserve the forage mass (Hu et al., 2009). The most common homofermentative inoculant is *Lactobacillus plantarum* (Koc et al., 2017). In order to improve aerobic stability, heterofermentative LAB such as *Lactobacillus buchneri* was developed as a silage inoculant. *Lactobacillus buchneri* improves aerobic stability by fermenting lactic acid to acetic acid and 1,2-propanediol (Elferink et al., 2001). However, how the homofermentative or heterofermentative LAB affect the bacterial community and metabolites in whole crop corn silage is unclear. Therefore, modulation of bacterial community and metabolome in corn silage, by inoculating *Lactobacillus plantarum* and *Lactobacillus buchneri*, were profiled in the present study.

## Materials and Methods

### Silage Making

The whole crop corn (*Zea mays* L.) was mowed with a precision chop harvester at a 234.6 g DM kg^-1^ of fresh forage. The pH of raw corn was 5.08. The epiphytic LAB, mold and yeast in the fresh corn were 7.08, 5.66, and 6.56 log_10_ cfu g^-1^, respectively. Vacuumed silos of whole crop corn (vacuum-sealing polyethylene plastic bags packed with approximately 300 g of fresh forage) were individually prepared for each of the following treatments: (a) untreated (control), (b) *L. plantarum*, and (c) *L. buchneri*. The application rate of each inoculant into the fresh forage was 1 × 10^6^ cfu g^-1^ FM, and an equal volume of distilled water was sprayed onto the fresh corn for the control group. The silos were then stored at ambient temperature (22–25°C) in dark conditions and sampled at 90 days of fermentation.

### Characteristics of Fresh Forage and Silage

A 20 g fresh sample was put in a juice exactor and squeezed with 180 mL distilled water for 30 s at a high speed, then filtered via medical gauze with four layers. The filtrate pH was measured with a glass electrode pH meter immediately. A portion of the filtrate of each sample was acidulated with H_2_SO_4_ (7.14 mol L^-1^) and filtered with a 0.45-μm dialyzer. Lactic acid, acetic acid, propionic acid, and butyric acid were analyzed by High Performance Liquid Chromatography (HPLC), (KC-811 column, Shodex; Shimadzu: Japan; oven temperature 50°C; flow rate 1 mL min^-1^; SPD 210 nm). Enumeration of LAB, yeast and mold in fresh corn were detected according to the methods described by Reich and Kung (2010). Briefly, samples (10 g) were homogenized in 100 mL of sterile Ringer’s solution (Oxide BR52) for 1 min and serially diluted (10-fold). The number of LAB was detected on spread plates using Rogosa agar (Oxoid CM627, Oxoid, Basingstoke, United Kingdom) and incubated at 37°C for 48–72 h. Yeast and mold were determined by pour plating serial 10-fold dilutions of water extracts on malt extract agar (Oxoid CM0059) that had been acidified with lactic acid (concentration of 850 g kg^-1^ added at 50 g kg^-1^, vol/vol). Plates were incubated at 32°C for 48–72 h. Colonies were counted from plates where appropriate dilutions yielded 30–300 colonies.

### SMRT Analysis of Bacteria Composition

Fresh and ensiled corn silages of each treatment were sampled for total bacteria DNA extraction. Total DNA was extracted using a DNA isolation kit (Tiangen, DP302-02, Tiangen, China) according to the manufacturer’s specification. The quality of extracted DNA samples was evaluated by 1% agarose gel electrophoresis and spectrophotometry. All extracted DNA samples were stored at -20°C for further analysis. The PCR amplification of the full-length 16S rRNA gene for SMRT sequencing was carried out with the forward primer 27F (5′-GAGAGTTTGATCCTGGCTCAG-3′) and reverse primer 1492R (5′-TACCTTGTTACGACTT-3′). The two primers contained a set of 16-nucleotide barcodes. The PCR program was as follows: 95°C for 3 min, 25 cycles of 98°C for 20 s, 57°C for 30 s and 72°C for 90 s, with a final extension of 72°C for 2 min.

The 16S rRNA library was built with a Pacific Biosciences Template Prep Kit. Sequencing of the amplicons was performed on a PacBio Sequel instrument (Pacific Biosciences, Menlo Park, CA, United States). The quality control for PCR amplifications and sequences pre-processing were performed as described by Mosher et al. (2013). Raw data was extracted and filtered with the Circular Consensus Sequencing (CCS) software of the SMRT Link (minfullpass = 3, polish minPredictedAccuacy = 0.8, minLength = 500) to obtain the Raw CCS Reads. The barcode reads of every sample were recognited with Lima^1^ to acquire Raw CCS. Whereafter the CCS (accuracy above 99%) of each sample was tested and chimeric reads were removed with UCHIME^2^ to acquire the optimized sequence. Subsequently, representative sequence was compared using the Mothur^3^ software with the Silva (Release 128^4^) database (classified at a bootstrap threshold of 0.9) to gain classified information (Quast et al., 2013). The Shannon-Wiener, Simpson’s diversity, Chao1 and rarefaction estimators were calculated to evaluate the alpha diversity.

### Metabolite Profiling Analysis

Samples of fresh and ensiled forages (5 g ± 1 mg) were extracted with 20 mL extraction liquid (VMethanol:VE.A = 1:1) in EP tubes. Then 666 μL of adonitol (0.5 mg mL^-1^ stock in dH_2_O) as internal standard was added and vortex mixing for 30 s. The mixture was oscillated for 1 h, and then filtered with membrane (0.22 μm). The supernatant (0.6 mL) was dried with a vacuum concentrator without heating in a GC/MS glass vial, and then 80 μL of methoxyamine hydrochloride (20 mg mL^-1^ in pyridine) was added into each dried metabolite and incubated for 30 min at 80°C. The quality control (QC) samples consisted of partial extract (75 μL) from each sample. For derivatization, 100 μL of the BSTFA regent (containing 1% TMCS, vol/vol) was added into each sample, incubated for 1.5 h at 70°C. Finally, 10 μL of FAMEs (standard mixture of fatty acid methyl esters, C8-C16:1 mg mL^-1^; C18-C24:0.5 mg mL^-1^ in chloroform) was added into the QC sample after cooling to the room temperature. All samples were analyzed by a Agilent 7890 gas chromatograph system coupled with a Pegasus 4D time-of-flight mass spectrometer (GC-TOF-MS).

The GC-TOF-MS system used a DB-5MS capillary column coated with 5% diphenyl and cross-linked with 95% dimethylpolysiloxane (30 m × 250 μm inner diameter, 0.25 μm film thickness; J&W Scientific, Folsom, CA, United States). Samples (1 μL) were injected in split mode (split ratio 20:1), with helium used as the carrier gas at a flow rate of 1.0 mL min^-1^. The oven temperature ramp was as follows: initial temperature was 80°C for 1 min, then raised to 290°C at a rate of 10°C min^-1^, and finally kept at 290°C for 13 min. The injection, transfer line, and ion source temperatures were 280, 295, and 220°C, respectively. The energy was -70 eV in electron impact mode. The mass spectrometry data was acquired in full-scan mode with an m z^-1^ range of 50–600 at a rate of 10 spectra per second, after a solvent delay of 7.9 min.

Chroma TOF 4.3X software of LECO Corporation and the LECO-Fiehn Rtx5 database were used for raw peak exaction, data baseline filtration and calibration of the baseline, as well as peak alignment, deconvolution analysis, peak identification and integration of the peak area (Kind et al., 2009). Both the mass spectrum match and retention index match were considered in metabolites identification. Peaks with poor repeatability (<50% of QC samples or RSD >30%) in QC samples were removed (Dunn et al., 2011). The NIST^5^ and KEGG^6^ commercial databases were used to search for metabolites. The followed method was used to calculate the relative concentration of each detected metabolite. Briefly, with the fixed volume of sample injected into equipment, a peak area of each metabolite as its relative concentration in the sample was obtained. The relative concentrations of metabolites were calculated as the peak area rate of each metabolite and internal standard substance (adonitol).

### Statistical Analysis

All metabolite data was normalized using SIMCA software (version 14, Umetrics AB, Umea, Sweden) before hierarchical cluster analysis and principal component analysis (PCA). PCA and projections to latent structure-discriminant analysis (PLS-DA) models were tested for all samples. The OPLS-DA model was employed with first principal-component of VIP (variable importance in the projection) values (VIP > 1) combined with Student’s *T*-test (*P* < 0.05) to find differentially expressed metabolites.

## Results

### Fermentation Quality of Whole Crop Corn Silage

The fermentation characteristics of whole crop corn silage ensiled for 90 days are shown in Table 1. Corn silage inoculated with *L. buchneri* had a higher pH, and greater acetic acid but less lactic acid compared with *L. plantarum*-treated and control groups (*P* < 0.05). Silage treated with *L. buchneri* had higher aerobic stability than samples treated with *L. plantarum*, but there was no significant difference with control group.

**Table 1:** Fermentation characteristics of whole crop corn silage for 90 days.

	Treatment^1^		
Item^2^	Control	LP	LB
pH	3.66b	3.68b	3.74a
LA g/kg	223.1a	218.7a	175.4b
AA g/kg	45.9b	41.7b	54.7a
PA g/kg	15.8a	13.5b	13.6b
LAB log_10_ cfu/g FM	8.03b	7.81b	8.32a
Yeast log_10_ cfu/g FM	3.3a	0b	2.85a
Mold log_10_ cfu/g FM	0b	3a	0b
Aerobic stability h	136.5a	114b	121.5ab

*^*1*^LP, samples treated with *L. plantarum*; LB, samples treated with *L. buchneri*. ^*2*^LA, lactic acid; AA, acetic acid, PA, propionic acid; LAB, lactic acid bacteria. Means within the same line with different letters are significantly different (*P* < 0.05).*

### Metabolomic Profiles of Whole Crop Corn Silage

**FIGURE 1:**
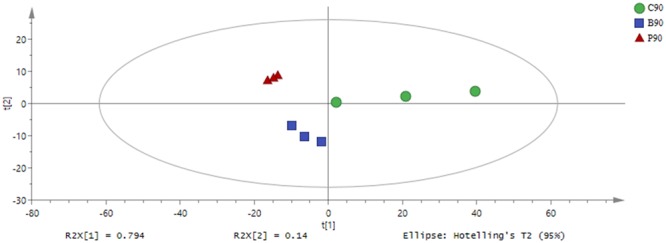
Principal component analysis (PCA) of metabolic profiles in whole crop corn silage inoculated without (green circle) or with *L. plantarum* (red triangle) or *L. buchneri* (blue square) (*n* = 3). Input data were the total mass of the signal integration area of each sample, and the signal integration area was normalized with method of internal standard normalization for each sample.

**FIGURE 2:**
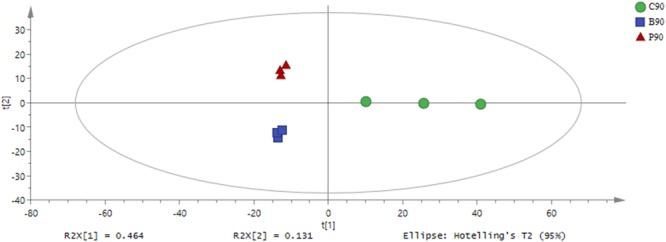
Partial least squares-discriminate analysis (PLS-DA) of metabolic profiles in whole crop corn silage inoculated without (green circle) or with *L. plantarum* (red triangle) or *L. buchneri* (blue square) (*n* = 3). Input data were the total mass of the signal integration area of each sample, and the signal integration area was normalized with method of internal standard normalization for each sample.

Based on the GC-TOF-MS of 12 samples, a total of 979 substances were detected, and 316 different metabolites were identified with their relative concentrations (Supplementary Table S1). According to PCA (Figure 1), the metabolites in *L. plantarum, L. buchneri* and control group samples were clearly separated by PC1, which represented 79.4% of variations among samples with different treatments. The altered metabolites in samples with or without inoculants were found from the first component of the PCA model, and the metabolites in *L. buchneri*-treated samples were separated by the second component of the PCA model. The contribution of metabolites to PC1 was dominated by lyxose, serine, oxoproline, proline, palmitic acid, succinic acid, and iminodiacetic acid (Supplementary Table S2). However, the application of multivariate analysis PLS-DA could be more useful for distinguishing control samples from *L. buchneri* and *L. plantarum*-treated samples (Figure 2).

The relative concentration and fold-changes of differentially expressed metabolites in whole crop corn silage with or without inoculants treatments are shown in Table 2. After 90 days of ensiling, samples treated with inoculants presented more amino acids, such as phenylalanine, lysine, tyrosine and glycine, than untreated samples. Compared to the control silage, up-accumulations of the phenolic acids of 4-hydroxycinnamic acid and 3,4-dihydroxycinnamic acid, the flavoring agent of gluconic lactone, and the organic acids of lauric acid, 3-hydroxypropionic acid, pentadecanoic acid, oxamic acid and isocitric acid were also observed in the inoculants treated silages. Inoculation of *L. buchneri* dramatically increased the relative concentrations of 2-hydroxybutanoic acid, saccharic acid, mannose, saccharic acid, 2-keto-L-gulonic acid, conduritol-β-epoxide, cholesterol and alpha-D-glucosamine-1-phosphate (a substance beneficial to health), in whole crop corn silage as compared to the *L. plantarum*-treated and control groups. Additionally, inoculation of *L. plantarum* remarkably increased the relative concentrations of cytosine, pyrogallol and tetrahydrocorticosterone in silages as compared to the control and samples inoculated with *L. buchneri*.

**Table 2:** Relative concentration and fold-changes in differential metabolites in whole crop corn silage with inoculation of *L. buchneri* or *L. plantarum* after 90 days of ensiling.

	Relative concentration^1^			Fold-changes^2^		
Metabolite name	Control	*L. buchneri*	*L. plantarum*	Log_2_ (B/C)	Log_2_ (P/C)	Log_2_(P/B)
Phenylalanine	0.000	0.014	0.054	–13.162	–15.104^∗∗^	–1.942^∗^
Lysine	0.000	0.325	0.741	–17.705	–18.894^∗∗^	–1.189
Tyrosine	0.000	0.087	0.217	–15.809^∗∗^	–17.125^∗^	–1.315^∗^
Glycine	0.000	0.006	0.006	–11.917^∗∗^	–12.057^∗∗^	–0.141
Oxamic acid	0.000	0.055	0.059	–14.701^∗∗^	–14.794^∗∗^	–0.093
2-Hydroxybutanoic acid	0.109	0.508	0.091	–2.218^∗^	0.264	2.482^∗^
3-Hydroxypropionic acid	0.000	0.010	0.010	–15.023^∗∗^	–15.012^∗∗^	0.011
2-Methylglutaric acid	0.018	0.001	0.003	4.883	2.858	–2.025^∗^
Lauric acid	0.000	0.021	0.026	–13.724^∗^	–14.069^∗^	–0.345
Isocitric acid	0.000	0.029	0.110	–14.231	–16.145^∗^	–1.914
4-Hydroxycinnamic acid	0.000	0.299	0.323	–17.586^∗∗^	–17.696^∗∗^	–0.110
Pentadecanoic acid	0.000	0.005	0.005	–11.594^∗∗^	–11.611^∗∗^	–0.016
3,4-Dihydroxycinnamic acid	0.000	0.028	0.029	–14.160^∗^	–14.197^∗^	–0.037
Quinic acid	0.011	0.007	0.000	0.647^∗^	15.988^∗∗^	15.341^∗∗^
Mannose	0.000	0.080	0.000	–15.677^∗^	–5.911	9.766^∗^
Melibiose	0.058	0.003	0.014	4.506^∗∗^	2.047^∗∗^	–2.459^∗^
Cellobiose	0.000	0.031	0.039	–14.304^∗∗^	–14.637^∗∗^	–0.333^∗^
Saccharic acid	0.025	0.217	0.035	–3.128^∗^	–0.513	2.615^∗^
2-Keto-L-gulonic acid	0.000	0.054	0.000	–15.127^∗∗^	3.169	18.297^∗∗^
Cytosine	0.000	0.000	0.158	3.211	–16.669^∗∗^	–19.880^∗∗^
Pyrogallol	0.000	0.000	0.008	3.211	–12.415^∗^	–15.625^∗^
Gluconic lactone	0.000	0.005	0.005	–11.753^∗∗^	–11.543^∗∗^	0.210^∗^
Alpha-D-glucosamine-1-phosphate	0.000	0.361	0.000	–17.859^∗^	3.169	21.028^∗^
4-Methyl-5-thiazoleethanol	0.000	0.017	0.004	–13.414^∗^	–11.367^∗^	2.047^∗^
Conduritol-β-epoxide	0.000	0.016	0.010	–13.323^∗^	–12.753^∗∗^	0.570
*N*-Acetyl-D-galactosamine	0.010	0.012	0.090	6.134	3.187	–2.947^∗^
Purine riboside	0.018	0.000	0.012	16.703^∗^	0.549	–16.154^∗∗^
Phytosphingosine	0.005	0.001	0.003	2.503^∗^	0.807	–1.696
Tetrahydrocorticosterone	0.000	0.001	0.002	–9.059	–10.500^∗∗^	–1.440
Cholesterol	0.000	0.001	0.000	–9.500^∗∗^	3.169	12.669^∗∗^

*^*1*^The relative concentration of each metabolite is an average of data from three biological replicates using GC-TOF-MS. ^*2*^The fold-changes were calculated using the formula log_2_(X/Y). X and Y refer different treatments: C, control; P, *Lactobacillus plantarum* treatment; B, *Lactobacillus buchneri* treatment; ^∗^0.001 < *P* < 0.05; ^∗∗^*P* < 0.001. The major metabolites were selected based on at least one of fold-changes [log_2_ (B/C), log_2_ (P/C), log_2_ (P/B)] contrast was statistically significant.*

Among the identified metabolites, some substances with biological functions in the whole crop corn silage were also detected (Table 3). Inoculants significantly decreased the relative concentration of phenylethylamine (PEA) as compared to the control silage. Some other metabolites with bacteriostatic activity, such as catechol, azelaic acid, ferulic acid, 3-phenyllactic acid, 3,4-dihydroxybenzoic acid, 4-hydroxybenzoic acid and glycolic acid, were found in the present whole crop corn silage although there were no significant differences in these metabolites among different treatments. Among those metabolites, catechol and ferulic acid have antioxidant properties, and 4-hydroxybutyrate has nervine activity. Linoleic acid with cholesterol lowering effects was detected at a higher relative concentration in the control silage compared to silages given inoculants. Myristic acid, a kind of flavoring agent, was also detected but no differences were found among treatments. The *L. plantarum*-treated samples contained less 4-aminobutyric acid than the control and samples treated with *L. buchneri*.

**Table 3:** Relative concentration and fold-changes in metabolites with biological functions in whole crop corn silage with inoculation of *L. buchneri* or *L. plantarum* after 90 days of ensiling.

	Relative concentration^1^			Fold-changes^2^		
Metabolite name	Control	*L. buchneri*	*L. plantarum*	Log_2_ (B/C)	Log_2_ (P/C)	Log_2_ (P/B)
Phenylethylamine	0.179	0.153	0.101	0.227^∗^	–0.831^∗∗^	–0.604^∗∗^
Catechol	0.037	0.020	0.024	5.336	–5.086	0.250
Linoleic acid	0.216	0.077	0.098	3.850^∗^	–3.510^∗^	0.340
Ferulic acid	0.465	0.196	0.242	2.369	–2.065	0.304
Myristic acid	0.042	0.024	0.028	0.801	–0.575	0.226
Azelaic acid	0.019	0.020	0.023	3.875	–3.709	0.167
Arachidonic acid	0.009	0.009	0.008	3.428	–3.628	–0.201^∗^
3-Phenyllactic acid	0.412	0.706	0.875	–0.779^∗^	1.089^∗^	0.309^∗^
3,4-Dihydroxybenzoic acid	0.409	0.247	0.085	0.728	–2.268^∗^	–1.539^∗∗^
4-Hydroxybenzoic acid	0.132	0.135	0.145	3.046	–2.943	0.103
4-Hydroxybutyrate	0.182	0.139	0.173	0.390	–0.072	0.318^∗^
4-Aminobutyric acid	1.446	1.194	0.576	0.276	–1.327^∗∗^	–1.051^∗^
Glycolic acid	0.002	0.001	0.001	0.646	–2.417	–1.770

*^*1*^The relative concentration of each metabolite is an average of data from three biological replicates using GC-TOF-MS. ^*2*^The fold-changes were calculated using the formula log_2_ (X/Y). X and Y refer different treatments: C, control; P, *Lactobacillus plantarum* treatment; B, *Lactobacillus buchneri* treatment; ^∗^0.001 < *P* < 0.05; ^∗∗^*P* < 0.001. The major metabolites were selected based on at least one of fold-changes [log_2_ (B/C), log_2_ (P/C), log_2_ (P/B)] contrast was statistically significant.*

### Bacterial Microbiota of Whole Crop Corn Silage

Based on SMRT sequencing of the full-length 16S rRNA gene in silage bacteria, an average of 22,240 CCS sequences were obtained from each sample. The Good’s coverage of samples showed that the sequence depth was adequate in the present study (Table 4). The α-diversity (Shannon index, Simpson index, Chao1 index), and alpha diversity index curves (Supplementary Figure S1) indicated high bacterial biodiversity in the present fermentation systems of whole crop corn silage.

**Table 4:** Sequence and bacterial diversity estimation of fresh forage and experimental treatment groups.

Sample ID	Average length (bp)	Observed species	Chao1	Shannon	Simpson	Goods coverage
FM.1	1464	1007	2741.3	8.06	0.99	0.88
FM.2	1469	1314	4485.7	8.13	0.99	0.88
FM.3	1460	892	1935.4	7.59	0.99	0.92
C90.1	1499	267	437.0	1.09	0.21	1.00
C90.2	1501	200	538.7	1.89	0.59	1.00
C90.3	1486	350	717.1	1.49	0.40	0.99
P90.1	1498	355	701.2	3.10	0.75	0.99
P90.2	1500	386	891.6	1.79	0.39	0.99
P90.3	1500	493	895.2	2.45	0.57	0.99
B90.1	1504	349	640.2	2.30	0.56	0.99
B90.2	1504	448	1103.1	2.49	0.63	0.99
B90.3	1490	235	398.5	2.19	0.57	1.00

*FM, fresh material; C, control; B, samples treated with *L. buchneri*; P, samples treated with *L. plantarum*; samples were fermented for 90 days. Goods coverage is calculated as C = 1 - (*s*/*n*), where *s* is the number of unique CCSs and *n* is the number of individuals in the sample. This index gives a relative measure of how well the sample represents the larger environment.*

**FIGURE 3:**
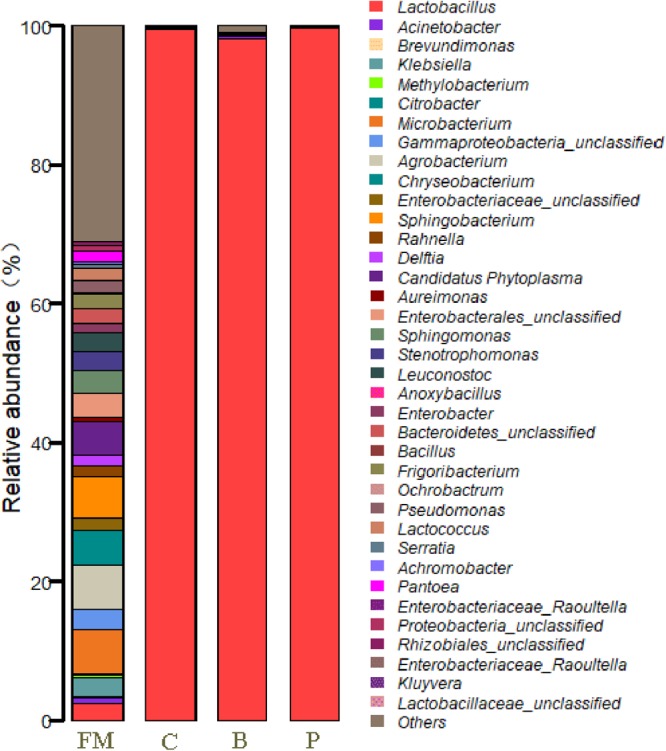
Relative abundances of the corn silage bacterial community before and after fermentation with different treatments for different ensiled times at genus level. FM, fresh material; C, control group; B, samples treated with *L. buchneri*; P, samples treated with L. plantarum.

**FIGURE 4:**
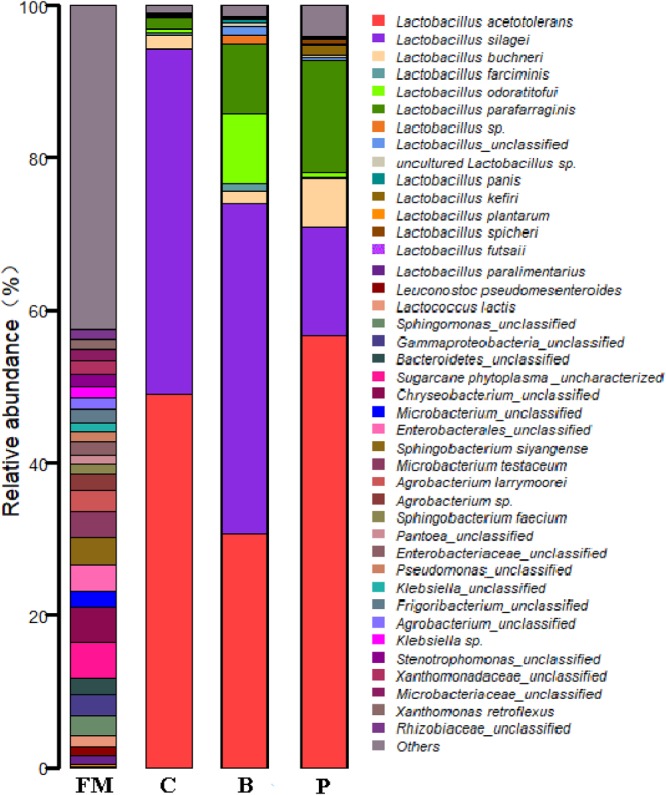
Relative abundances of the corn silage bacterial community before and after fermentation with different treatments for different ensiled times at species level. FM, fresh material; C, control group; B, samples treated with *L. buchneri*; P, samples treated with *L. plantarum*.

At the genus level, the epiphytic microflora of fresh corn was mainly comprised by *Agrobacterium, Microbacterium, Sphingobacterium, Chryseobacterium, Candidatus Phytoplasma*, unclassified *Enterobacterales*, unclassified *Gammaproteobacteria, Leuconostoc* (2.69%), *Klebsiella, Stenotrophomonas, Lactobacillus* (2.44%), *Frigoribacterium*, unclassified *Bacteroidetes* and others (Figure 3). After 90 days of ensiling, the dominated microflora was the genus *Lactobacillus* (>98%), regardless of treatments. At the species level (Figure 4), fresh corn exhibited the greatest species richness and was harbored by many undesirable bacteria such as unclassified *Chryseobacterium* (4.6%), *Sphingobacterium siyangense* (3.5%), unclassified *Enterobacterales* (3.5%), *Microbacterium testaceum* (3.5%), unclassified *Sphingomonas* (2.6%), and so on. Meanwhile, the epiphytic LAB on the fresh corn mainly consisted of *Lactococcus lactis* (1.58%), *Leuconostoc pseudomesenteroides* (1.13%), *Lactobacillus paralimentarius* (1.06%), *Lactobacillus plantarum* (0.37%) and *Lactobacillus farciminis* (0.03%). After fermentation for 90 days, *Lactobacillus* species such as *L. acetotolerans, L. silagei, L. parafarraginis, L. buchneri*, and *L. odoratitofui* dominated the bacterial community. Compared to control group, samples inoculated with *L. plantarum* increased the relative abundances of *L. acetotolerans, L. parafarraginis, L. buchneri*, and decreased the relative abundance of *L. silagei*. Meanwhile, inoculation of *L. buchneri* increased the relative abundances of *L. silagei, L. odoratitofui, L. parafarraginis, L. farciminis*, and diminished the relative abundance of *L. acetotolerans.*

### Correlations Between the Relative Abundance of Bacteria and Metabolites of Ensiled Whole Crop Corn

**FIGURE 5:**
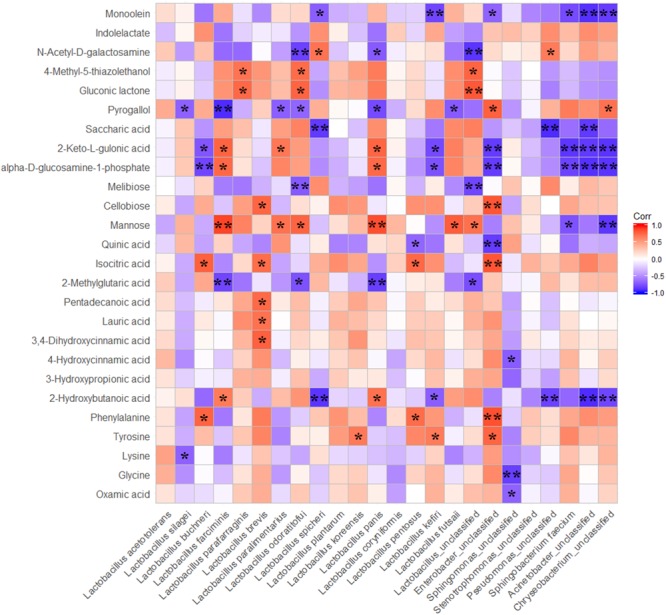
Spearman correlations between main bacteria species and differnentially presented metabolites. The differnentially presented metabolites during fermentation were screened by OPLS-DA; P-values are shown as ^∗^0.01 < *P* ≤ 0.05, ^∗∗^*P* ≤ 0.01.

Spearman correlations between the dominated bacteria and the metabolites that were differentially presented in ensiled samples within three treatments were calculated. This indicated perfect negative to perfect positive correlations (ranges from -1 to 1; *P*-values are shown as ^∗^0.01 < *P* ≤ 0.05, ^∗∗^*P* ≤ 0.01) for the 90-day fermented corn silages (Figure 5). Many of metabolites were positively correlated with LAB species, and were negatively correlated with undesirable bacteria presented during ensiling. Mannose was positively correlated with *L. farciminis, L. parafarraginis, L. odoratitofui, L. panis, L. futasaii* and unclassified *Lactobacillus*, but it was negatively correlated with unclassified *Sphingomonas* and unclassified *Chryseobacterium*. Generally, some end products of fermentation were positively correlated with some species of LAB but were negatively correlated with the other LAB species. For instance, 2-keto-L-gulonic acid and the health beneficial substance (alpha-D-glucosamine-1-phosphate) were positively correlated with *L. farciminis* and *L. panis*, whereas they were negatively correlated with *L. buchneri, L. kefiri* and undesirable bacteria.

**FIGURE 6:**
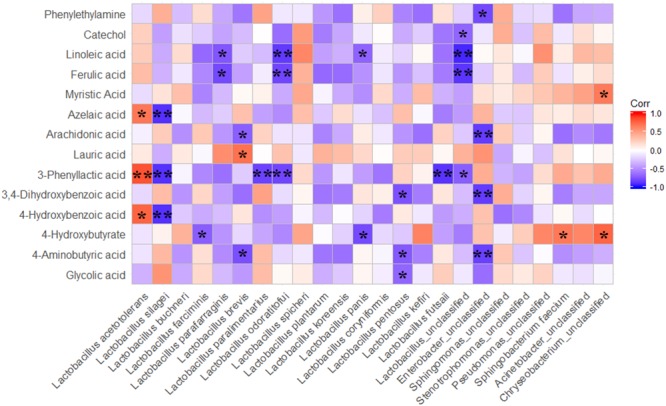
Spearman correlations between metabolites with biofunctional activity and main bacteria species. The differnentially presented metabolites during fermentation were screened by OPLS-DA; P-values are shown as ^∗^0.01 < *P* ≤ 0.05, ^∗∗^*P* ≤ 0.01.

The correlations between the dominant bacterial species and other metabolites with biological functions, which were not screened out as differential metabolites among treatments by using OPLS-DA analysis, were also analyzed by Spearman correlation analysis (Figure 6). The results exhibited that the *L. acetotolerans* positively correlated with 4-hydroxybutyrate, 3-phenyllactic acid and azelaic acid. Lauric acid positively correlated with *L. brevis*. In addition, most of metabolites with biological function were negatively correlated with LAB species. It is interesting that the correlation between metabolites and *L. acetotolerans* was diametrically opposite with the correlation between corresponding metabolites and *L. silagei*.

## Discussion

Ensiling is a complex process dominated by the epiphytic microbial community of LAB. Metabolomic and bacterial community analysis of silage fermentation system is proposed to have contributed important information on screening targeted LAB for modulating silage fermentation in order to make high-quality silage, and even for broadening silage function from nutritive value with perspective to being beneficial to animal health and welfare. This study, combining metabolomic and bacterial community analysis, for the first time revealed the characteristics of metabolome and bacterial community in whole crop corn silage inoculated by homofermentative *L. plantarum* and heterofermentative *L. buchneri*. Moreover, the present study also revealed that homofermentative and heterofermentative inoculants directed the fermentation process of whole crop corn silage with different manners.

### Effects of Inoculants on Fermentation Characteristics in Whole Crop Corn Silage

Lactic acid bacteria are often inoculated into forage crops at ensiling for improving silage fermentation. Theoretical homofermentative LAB can rapidly reduce the pH of silage and help to preserve more forage mass, while heterofermentative LAB can produce much more acetic acid to improve aerobic stability. Fermentation parameters in the present study verified the theory. Compared with *L. plantarum*-inoculated samples, *L. buchneri*-treated samples had a higher pH and concentration of acetic acid, better aerobic stability and a lower concentration of lactic acid. There was no significance between the *L. plantarum*-inoculated and untreated group on parameters of fermentation characteristics. This study showed a similar effect of inoculation of *L. plantarum* and *L. buchneri* on forage fermentation quality to previous research (Ranjit and Kung, 2000; Weinberg et al., 2002).

### Effects of Inoculants on Metabolomic Profiles in Whole Crop Corn Silage

Metabolome profiles of the present whole crop corn silage indicated that inoculation of the homofermentative *L. plantarum* or heterofermentative *L. buchneri* differently modulated the metabolite composition pattern during the ensiling. Over the past two decades, researches on metabolites in ensiled forages were mainly focused on the organic acids and 1, 2-propanediol in order to evaluate fermentation quality and aerobic stability of silages. Our results showed that samples treated with inoculants resulted in an up-accumulation of some free amino acids, which agrees with the results of previous studies on alfalfa silage (Guo et al., 2018). However, there was a big difference in metabolite types and compositions between the present whole crop corn silage and alfalfa silage previously studied (Guo et al., 2018). It might be due to there being a different microbial community present or due to the different fermentation that occurred in the various ensiled forage species. Some metabolites with antimicrobial activity were detected in the present study, such as 4-hydroxycinnamic acid, 3,4-dihydroxycinnamic acid, catechol, azelaic acid, 3-phenyllactic acid, 3,4-dihydroxybenzoic acid, 4-hydroxybenzoic acid, ferulic acid and glycolic acid. Among these metabolites, catechol, azelaic acid, 3-phenyllactic acid, 4-hydroxybenzoic acid and ferulic acid had also been detected in grass silage, and higher catechol, azelaic acid, 3-phenyllactic acid and ferulic acid levels were observed in grass silages inoculated with LAB strains compared to the control silage (Broberg et al., 2007). In the present study, except for 4-hydroxycinnamic acid, 3,4-dihydroxycinnamic acid and 3-phenyllactic acid, no difference was observed in the other antimicrobial compounds between the control silage and inoculants treated silages. It might be because these metabolites with antimicrobial activity are produced by different bacteria, or because the interaction or competition with different microbes affected the end accumulation of these metabolites.

Some substances with other biological functions were detected in the ensiled whole crop corn silages after 90 days of fermentation. Catechol, pyrogallol and ferulic acid with antioxidant property, 4-hydroxybutyrate with nervine activity, and linoleic acid with cholesterol lowering effects, were discovered in the present study. In addition, a flavoring agent of myristic acid and a depression mitigation substance of PEA were also found in the whole crop corn silage. Previously, these metabolites had not been paid close attention to, nor their biofunctional activity in silages for animal health and welfare. As a non-protein amino acid in animals, 4-aminobutyric acid is a major inhibitory neurotransmitter and can decrease blood pressure (Pouliot-Mathieu et al., 2013). Our previous study showed that *L. buchneri* up-accumulated 4-aminobutyric acid in ensiled alfalfa (Guo et al., 2018), but the results from the present study indicated that *L. plantarum* resulted in a down-accumulation of 4-aminobutyric acid, and there was no difference in 4-aminobutyric acid between the control and *L. buchneri*-treated silages. These results suggested that inoculants had different modulation manners in different forages with various epiphytic microorganisms at ensiling. However, numerous functional ingredients and flavoring agents were detected in the whole crop corn silages in the present study, which suggested that metabolomic analysis is an effective way to comprehensively evaluate the fermentative, nutritive, and functional profiles of ensiled forages. As for the detection method, however, we did not optimize the oven temperature ramp for metabolite analysis through the GC-TOF-MS system. Thus, it is necessary to explore an optimal temperature program suitable for analyzing metabolites in the fermentative system of silage.

### Effects of Inoculants on Bacterial Microbiota in Whole Crop Corn Silage

It is well established that natural fermentation of forges depends on epiphytic microbial community especially LAB in anaerobic environment (Jones, 1991). In addition, various microbial community and succession were found in different pro- and after-ensiled forages (Parvin et al., 2010). Therefore, microbial community composition plays a crucial role in fermentation of ensiled forages, and it is necessary to know the community composition to understand the complex process of ensiling. In the present study, *Agrobacterium, Microbacterium, Sphingobacterium, Chryseobacterium, Candidatus Phytoplasma*, unclassified *Enterobacterales*, unclassified *Gammaproteobacteria* and a small proportion of LAB (2.69% *Leuconostoc* and 2.44% *Lactobacillus*) dominated the microbial composition in fresh whole crop corn before ensiling. While, the results of a previous report indicated that *Leuconostocaceae, Acetobacteraceae, Enterobacteriaceae, Moraxellaceae*, and *Lactobacillaceae* were the dominant bacteria in fresh whole crop corn before ensiling (Gharechahi et al., 2017). The differences between studies suggest that colonization of plant surfaces by bacteria depends on many factors including plant species, climate, period of duration, geographical location, solar radiation intensity and the type of fertilizer used (Cai et al., 1999a; Pang et al., 2011; Mcgarvey et al., 2013). After fermentation, most of the undesirable microorganisms were inhibited at anaerobic condition, which was confirmed well in the present study because *Lactobacillus* occupied over 98% in silages stored for 90 days. Furthermore, organic acids and metabolites with antimicrobial activity produced by LAB inhibited the undesirable microbes during ensiling. The present study indicated that *L. acetotolerans, L. silagei, L. buchneri, L. odoratitofui, L. farciminis* and *L. parafarraginis* were the dominant microbial flora in the whole crop corn silage after 90 days of fermentation. Based on the previous studies, *L. acetotolerans* was detected in traditional pot fermentation of rice vinegar (Entani et al., 1986; Haruta et al., 2006); *L. farciminis* was isolated from fermenting mushroom, which exhibited potential for a bacteriocin and antibiotic assay (Halami et al., 2000), or was isolated from ensiled corn stover with feruloyl esterase (FAE) activities (Xu et al., 2017a); *L. Paralimentarius* was isolated from sourdough (Cai et al., 1999b); *L. odoratitofui* and *L. silagei* were isolated from orchardgrass silage or stinky tofu brine (Chao et al., 2010; Tohno et al., 2013); and *L. parafarraginis* was isolated from sudan-grass silage or corn stover silage (Liu et al., 2014; Xu et al., 2017b). However, these dominated LAB species had not been previously reported in the whole crop corn silage. It has been verified that *L. acetotolerans is* a homofermentative species resistant to high concentrations of acetic acid, while some physiological properties differ from other homofermentative species of the genus *Lactobacillus* (Entani et al., 1986). In the present study, the *L. buchneri*-treated group had a much greater abundance of *L. parafarraginis*. *Lactobacillus parafarraginis* has been reported to improve the aerobic stability of silages (Liu et al., 2014), which provided further evidence that inoculation of *L. buchneri* can improve aerobic stability of silage.

As for modulation of the bacterial community in the whole crop corn silage by inoculants, different microbial communities were observed in the control silage and silages treated with the two inoculants after 90 days of fermentation. In the control silage, *L. acetotolerans* and *L. silagei* were the most dominant LAB species, as they accounted for 93.2% of the total bacterial community: the relative abundances of *L. acetotolerans* and *L. silagei* were 48.9% and 45.3%, respectively. The microbial community in the *L. plantarum* treated silage was mainly comprised of *L. acetotolerans* (56.6%), *L. parafarraginis* (14.7%), *L. silagei* (14.2%), *L. buchneri* (6.4%), *L. kefiri* (1.3%), and *L. odoratitofui* (0.7%). In the *L. buchneri* inoculated silage, the microbial community mainly consisted of *L. silagei* (43.4%), *L. acetotolerans* (30.7%), *L. odoratitofui* (9.3%), *L. parafarraginis* (9.1%), *L. buchneri* (1.4%), and *L. farciminis* (0.97%). These results indicated that inoculation of *L. plantarum* markedly increased the relative abundances of *L. acetotolerans, L. buchneri*, and *L. parafarraginis*, but resulted in a considerable decline in the proportion of *L. silagei*; whereas an obvious decrease in *L. acetotolerans* and increases in *L. odoratitofui* and *L. farciminis* were observed in the *L. buchneri* inoculated silage. According to a previous study, *L. buchneri* inoculation did not alter the indigenous bacterial community in whole crop corn silage, and the *L. buchneri* was detected only as additions with DGGE (Li and Nishino, 2011b). Parvin et al. (2010) also studied the bacterial communities in whole crop maize silage inoculated with *L. buchneri* and *L. plantarum* by DGGE The results indicated that the bands of *L. lactic, Weissella paramesenteroides* and *Pediococcus pentosaceus* became faint after treatment with *L. plantarum*, and the band of *L. lactic* was extensive in *L. buchneri*-treated silage. Inoculation of *L. plantarum* promoted the growth of *L. buchneri* in the present whole crop corn silage, however, lower *L. buchneri* was observed in *L. buchneri* inoculated silage, which supports our previous results on alfalfa silage (Guo et al., 2018). Therefore, heterofermentative *L. buchneri* and homofermentative *L. plantarum* both modulated silage fermentation in different ways as shown by the various microbial communities and metabolite composition in ensiled forage.

### Correlations Between Main Bacterial Species and Metabolites of Whole Crop Corn Silage

To explore the correlations between main bacteria and the main metabolites, Spearman correlation analysis was performed. The results showed that metabolites were positively correlated with LAB species and were negatively correlated with undesirable bacteria presented during ensiling. These results suggested that LAB were more competitive than the undesirable bacteria in the airtight environment of silos. However, some end products of fermentation were positively correlated with some of LAB species but were negatively correlated with the other LAB species. This indicated that competition and synergy were concurrent between different species of bacteria during ensiling. Additionally, crop characteristics of dry matter content, sugar content, and sugar composition in combination with lactic acid bacterial properties, such as acid and osmotolerance, and substrate utilization, will decisively influence the competitiveness of the lactic acid bacterial flora during silage fermentation (Jones, 1991). After fermentation for 90 days, abundances of *L. acetotolerans* and *L. silagei* in total reached 70%. However, correlations between the two dominant species and those differentially presented metabolites were not significant, with the exception of the negative correlation between the two strains and the metabolites of pyrogallol and lysine. It suggested that the differences of metabolites between treatments probably did not result from the dominant species. However, there was a considerable difference in the abundance of *L. buchneri* and *L. odoratitofui* among the treatments, and a positive correlation between these two LAB species and some metabolites was also observed. Based on the positive correlation between *L. buchneri* and phenylalanine and the up-accumulation of phenylalanine in *L. plantarum*-treated samples, it can be inferred that *L. buchneri* could improve phenylalanine in ensiled forage. In addition, the up-accumulations of phenylalanine, lysine and tyrosine were also detected in the present whole crop corn silage inoculated with *L. buchneri*. All these results indicated that *L. buchneri* could produce some amino acids during fermentation of ensiled forages as it was suggested by our previous research on alfalfa silage (Guo et al., 2018). Higher abundance of *L. odoratitofui* in *L. buchneri*-treated samples was detected, which was positively correlated with mannose, gluconic lactone and 4-methyl-5-thiazoleethanol and was negatively correlated with 2-methylglutaric acid, melibiose, pyrogallol and *N*-acetyl-D-galactosamine. The results indicated that mannose, gluconic lactone and 4-methyl-5-thiazoleethanol were contributed by *L. odoratitofui*. Although a small proportion of *L. farciminis* was observed in *L. buchneri*-treated samples, the positive correlation between this species and metabolites showed that 2-hydroxybutanoic acid, mannose, 2-keto-L-gulonic acid and the health beneficial substance of alpha-D-glucosamine-1-phosphate were contributed by *L. farciminis*. Moreover, positive correlations between some observed metabolites and the minimal proportions of *L. brevis, L. paralimentarius, L. koreensis, L. panis, L. pentosus, L. kefiri* and *L. futsaii* indicated that these LAB species also played considerable roles in accumulation of those metabolites, in spite of their minute amounts. No significant correlation was observed between *L. plantarum* and all the detected metabolites because only a small proportion of *L. plantarum* was observed in *L. buchneri*-treated samples after 90 days of fermentation.

As for the correlations between bacterial species and metabolites with biofunctions, the result indicated that the correlation between the metabolites (4-hydroxybenzoic acid, 3-phenyllactic acid, azelaic acid) and *L. acetotolerans* was diametrically opposite with the correlation between the corresponding metabolites and *L. silagei*. The *L. plantarum*-inoculated samples presented a higher abundance of *L. acetotolerans* and a lower abundance of *L. silagei*, however, contrary results were observed in *L. buchneri*-treated samples. Correspondingly, *L. plantarum*-treated samples showed higher relative concentrations of 3-phenyllactic acid, 4-hydroxybutyrate and azelaic acid compared to the *L. buchneri*-treated samples. These results suggested that the antimicrobial compounds of 4-hydroxybenzoic acid, 3-phenyllactic acid and azelaic acid were probably produced by *L. acetotolerans*. Metabolites with biofunctions negatively correlated with LAB species, which might be a result of the concentrations in extracts of samples being too low to be detected accurately, yet still high enough to affect other microorganisms and animal health and welfare. However, correlation is not causation (Geer, 2011), it is based on statistics and correlates parameters to obtain a result that represents nothing more than speculation. It should also be noted that lack of a significant correlation between some bacterial taxa and metabolites in the correlation analysis does not necessarily mean those bacterial taxa or metabolites are not related. Furthermore, the functional features of a small number of species can also have a large impact on community structure and ecosystem functioning (Kruger Ben Shabat et al., 2016). Even so, the correlations between LAB and the metabolites with biological functions in the ensiled whole crop corn silage provided important information on screening targeted LAB for modulating silage fermentation in order to make high quality silage, and even for extending silage function from nutritive value with perspective to being beneficial to animal health and welfare. Therefore, the screening and application of inoculants for silage with secretion of biological substances, such as amino acids, small peptides, flavor agents, bacteriocin and other antibacterial agents (vitamins, polysaccharides, etc.), will be another prosperous research field on silage for future study.

## Conclusion

Inoculation of *L. plantarum* and *L. buchneri* altered the microbial composition and fermentative metabolites in ensiled whole crop corn silage in very different ways. Heterofermentative *L. buchneri* resulted in an up-accumulation of 2-hydroxybutanoic acid, saccharic acid, mannose, saccharic acid, 2-keto-L-gulonic acid and alpha-D-glucosamine-1-phosphate (a substance beneficial to health), and increased the abundances of the *L. silagei, L. odoratitofui, L. parafarraginis, L. farciminis* but diminished the abundance of *L. acetotolerans.* Homofermentative *L. plantarum* markedly increased the relative concentrations of pyrogallol and tetrahydrocorticosterone and increased the abundances of *L. acetotolerans, L. parafarraginis, L. buchneri*, but decreased the abundance of *L. silagei*. The correlations of metabolites and bacterial species revealed that dominant species *L. acetotolerans* and *L. silagei* did not result in the differences of metabolites. The LAB species which were markedly modulated by the two types of inoculants were closely correlated to some of metabolites. Thus, correlations between metabolites and bacterial species can provide important scientific information on screening targeted LAB for modulation of silage fermentation. Some new metabolites such as substances with bacteriostatic activity, antioxidant properties, nervine activities, cholesterol lowering effects or other flavoring agents were detected in the present whole crop corn silage. Some LAB species such as *L. acetotolerans, L. farciminis, L. Paralimentarius, L. odoratitofui* and *L. silagei* were discovered in the present whole crop corn silage. Therefore, profiling of silage microbiome and metabolome can improve our current understanding of the biological process underlying silage formation, and will be helpful to re-evaluate silage, with respect to nutritive value and fermentation quality but also to animal health and welfare.

## Author Contributions

DX and XG designed the study and wrote the manuscript. DX, WK, WD, FL, and PZ performed the experiments. DX and XG analyzed the data. All authors reviewed the manuscript.

## Conflict of Interest Statement

The authors declare that the research was conducted in the absence of any commercial or financial relationships that could be construed as a potential conflict of interest.

## Footnotes

1 https://github.com/PacificBiosciences/barcoding

2 http://drive5.com/uchime

3 https://mothur.org/wiki/Classify.seqs

4 http://www.arb-silva.de

5 http://www.nist.gov/index.html

6 http://www.genome.jp/kegg/

## Supplementary Material

FIGURE S1 Alpha diversity index curves showing the diversity of taxa present in each sample [rarefaction curve (left) and rank abundance curve (right)]. D0.1–D0.3, whole crop corn before ensiling.

TABLE S1 The relative concentration of 316 indentified metabolites in the control, *Lactobacillus plantarum*- and *Lactobacillus buchneri*-inoculated silages with triplicate for each treatment.

TABLE S2 The contribution of 316 indentified metabolites to the first principal component (PC1) and standard error.
