# Supplementary figures and images for: Modulation of Metabolome and Bacterial Community in Whole Crop Corn Silage by Inoculating Homofermentative Lactobacillus plantarum and Heterofermentative Lactobacillus buchneri
Source: Front Microbiol. 2019 Jan 23;9:3299. doi: 10.3389/fmicb.2018.03299 (PMC6352740; doi:10.3389/fmicb.2018.03299)

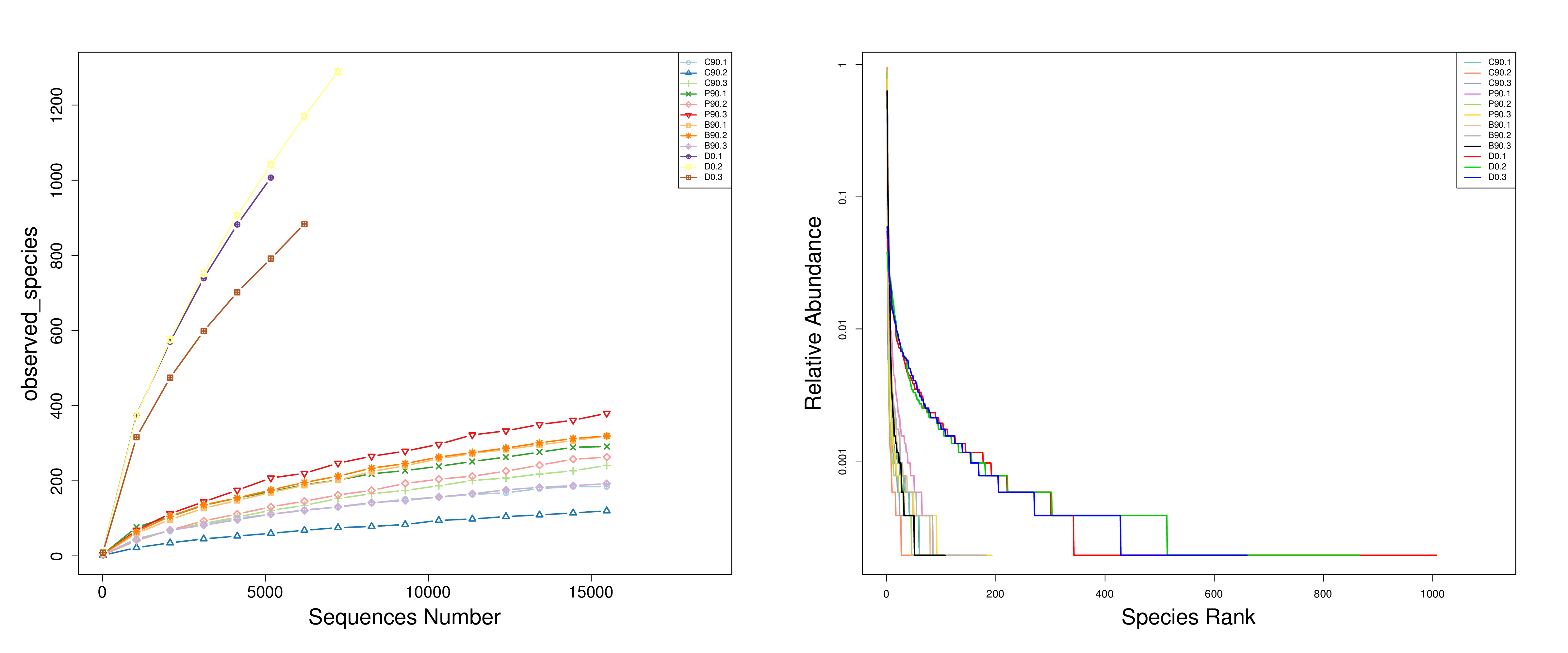

Supplement: FIGURE S1 — Alpha diversity index curves showing the diversity of taxa present in each sample [rarefaction curve (left) and rank abundance curve (right)]. D0.1–D0.3, whole crop corn before ensiling. [file Image_1.TIF]
