# Supplementary material for: Modulation of Metabolome and Bacterial Community in Whole Crop Corn Silage by Inoculating Homofermentative Lactobacillus plantarum and Heterofermentative Lactobacillus buchneri
Source: Front Microbiol. 2019 Jan 23;9:3299. doi: 10.3389/fmicb.2018.03299 (PMC6352740; doi:10.3389/fmicb.2018.03299)
Supplement: TABLE S1 — The relative concentration of 316 indentified metabolites in the control, Lactobacillus plantarum- and Lactobacillus buchneri-inoculated silages with triplicate for each treatment. [file Table_1.docx]

**Modulation of metabolome and bacterial community in whole crop corn silage by inoculating homofermentative *Lactobacillus plantarum* and heterofermentative *Lactobacillus buchneri***

**Running title: Metabolome and microbiota in silage**

Dongmei Xu^1,2^, Wencan Ke^1,2^, Wurong Ding^1,2^, Fuhou Li^2,3^, Ping Zhang ^1,2^, Xusheng Guo^1,2^*****

^1^State Key Laboratory of Grassland and Agro-ecosystems, School of Life Sciences, Lanzhou University, Lanzhou 730000, PR China, guoxsh07@lzu.edu.cn

^2^Probiotics and Biological Feed Research Center, Lanzhou University, Lanzhou 730000, PR China

^3^Stay Key Laboratory of Grassland Agro-ecosystems, College of Pastoral Agriculture Science and Technology, Lanzhou University, Lanzhou 730020, China.

**TABLE S1** The relative concentration of 316 indentified metabolites in the control, *Lactobacillus plantarum*- and *Lactobacillus buchneri*-inoculated silages with triplicate for each treatment^1^

| Peak | Control_1 | Control_2 | Control_3 | *L.buchneri*_1 | *L.buchneri*_2 | *L.buchneri*_3 | *L.plantarum*_1 | *L.plantarum*_2 | *L.plantarum*_3 |
| --- | --- | --- | --- | --- | --- | --- | --- | --- | --- |
| Pyruvic acid | 0.038676 | 3.97E-06 | 0.008562 | 1.81E-07 | 0.013086 | 1.55E-07 | 0.004755 | 0.007831 | 0.007177 |
| Lactic acid | 6.782539 | 2.511249 | 4.646894 | 7.369417 | 6.502369 | 5.172594 | 3.220081 | 4.37211 | 5.657825 |
| Glycolic acid | 0.003661 | 0.000953 | 0.002307 | 0.001277 | 0.001869 | 0.001275 | 1.71E-07 | 0.001296 | 1.61E-07 |
| Alanine | 4.2E-07 | 0.000694 | 0.017249 | 1.81E-07 | 1.56E-07 | 0.017681 | 0.000383 | 1.74E-07 | 0.013944 |
| 2-keto-isovaleric acid | 0.001439 | 3.97E-06 | 0.000644 | 0.000527 | 0.000445 | 1.55E-07 | 1.71E-07 | 0.000464 | 1.61E-07 |
| Hydroxylamine | 0.742433 | 21.59286 | 1.039333 | 0.463602 | 0.31161 | 0.666143 | 0.473082 | 0.748826 | 0.478585 |
| 2-Hydroxybutanoic acid | 0.078771 | 0.139698 | 0.109235 | 0.402691 | 0.755093 | 0.367047 | 0.068947 | 0.066661 | 0.137308 |
| Sarcosine | 0.292715 | 0.156222 | 0.224468 | 0.161388 | 0.126806 | 0.155406 | 0.110093 | 0.125054 | 0.145595 |
| 3-Hydroxypropionic acid | 4.2E-07 | 1.75E-07 | 2.98E-07 | 0.011065 | 0.009801 | 0.00886 | 0.009067 | 0.010001 | 0.010437 |
| 3-Hydroxybutyric acid | 3.064081 | 25.93079 | 1.28749 | 0.943004 | 0.823491 | 1.188681 | 1.040342 | 1.249909 | 1.251014 |
| 3-Hydroxypyridine | 0.079457 | 0.734995 | 1.75E-07 | 1.81E-07 | 0.02909 | 1.55E-07 | 0.029366 | 0.030921 | 0.029628 |
| Sulfuric acid | 0.086048 | 0.063112 | 0.07458 | 0.309061 | 0.180961 | 0.173056 | 0.194699 | 0.243889 | 0.197545 |
| 4-Aminobutyric acid | 1.461552 | 1.430645 | 1.446099 | 1.33361 | 1.336735 | 0.913012 | 0.527633 | 0.601584 | 0.599776 |
| Benzyl alcohol | 4.2E-07 | 3.97E-06 | 1.75E-07 | 1.81E-07 | 1.56E-07 | 0.000295 | 1.71E-07 | 0.000368 | 0.000261 |
| N-Methyl-DL-alanine | 0.00579 | 3.97E-06 | 1.75E-07 | 1.81E-07 | 1.56E-07 | 0.002421 | 0.002445 | 0.002533 | 0.002537 |
| 2-amino-2-methylpropane-1,3-diol | 0.296482 | 8.861526 | 0.422165 | 0.139013 | 0.091068 | 0.224151 | 0.179273 | 0.323367 | 0.180526 |
| Lactamide | 4.2E-07 | 0.452877 | 0.019833 | 0.019329 | 0.01662 | 0.016239 | 0.017562 | 0.021695 | 0.019959 |
| 2-Amino-3-methyl-1-butanol | 0.034269 | 0.358404 | 1.75E-07 | 0.015013 | 0.014211 | 0.01356 | 0.013669 | 1.74E-07 | 0.014423 |
| O-methylthreonine | 0.087439 | 2.087818 | 0.248652 | 3.835292 | 5.452238 | 3.151068 | 1.242956 | 1.085382 | 1.541542 |
| Succinate semialdehyde | 0.009685 | 0.116186 | 0.004529 | 0.003246 | 0.003394 | 0.002784 | 0.005527 | 0.00528 | 0.004992 |
| Malonic acid | 0.015802 | 0.154892 | 1.75E-07 | 0.00609 | 0.00633 | 0.007188 | 0.007509 | 0.007486 | 0.006617 |
| Norleucine | 0.006929 | 3.97E-06 | 0.002897 | 1.81E-07 | 1.56E-07 | 1.55E-07 | 1.71E-07 | 1.74E-07 | 0.003493 |
| Methylmalonic acid | 0.17593 | 2.384998 | 1.75E-07 | 0.023265 | 0.013471 | 0.051278 | 0.05965 | 0.060003 | 0.041985 |
| alpha-Ketoisocaproic acid | 0.169951 | 1.727594 | 0.083592 | 0.06852 | 0.06772 | 0.06207 | 0.053291 | 0.08394 | 0.074846 |
| Carnitine | 0.003582 | 0.083138 | 1.75E-07 | 0.002422 | 0.002563 | 0.003559 | 0.001749 | 0.004095 | 0.002797 |
| Valine | 4.2E-07 | 35.69044 | 1.930523 | 2.294273 | 1.78904 | 2.041657 | 1.75825 | 1.903602 | 1.834119 |
| Canavanine degr prod | 0.027718 | 0.468852 | 0.024603 | 0.016554 | 0.016231 | 0.013062 | 0.015082 | 0.024341 | 0.022241 |
| Pyrophosphate | 0.032371 | 0.681169 | 0.027033 | 0.022106 | 0.019401 | 0.019439 | 0.025119 | 0.037232 | 0.035252 |
| 2-Butyne-1,4-diol | 0.004787 | 0.05098 | 0.002493 | 0.001895 | 0.001793 | 0.001685 | 1.71E-07 | 0.002286 | 0.001977 |
| Guaiacol | 4.2E-07 | 3.97E-06 | 1.75E-07 | 0.001677 | 0.001731 | 0.001806 | 0.001777 | 1.74E-07 | 0.001872 |
| 4-Hydroxybutyrate | 0.218403 | 0.145981 | 0.182192 | 0.138691 | 0.152037 | 0.126483 | 0.166596 | 0.182898 | 0.170552 |
| 2-Ketoadipate | 0.028679 | 0.010281 | 0.01948 | 0.01109 | 0.010514 | 0.009299 | 0.00882 | 0.011306 | 0.010656 |
| Dihydroxyacetone | 0.020708 | 0.339631 | 0.017344 | 0.022931 | 0.021223 | 0.026412 | 0.01879 | 0.025488 | 0.025533 |
| Benzoic acid | 0.045699 | 0.022885 | 0.034292 | 0.01724 | 0.017778 | 0.017509 | 0.019252 | 0.02054 | 0.017421 |
| Oxamic acid | 3.97E-06 | 1.75E-07 | 2.07E-06 | 0.058929 | 0.056618 | 0.049809 | 0.055606 | 0.064576 | 0.056223 |
| Ethanolamine | 4.2E-07 | 70.58019 | 3.261107 | 3.185756 | 2.866056 | 2.823344 | 3.064224 | 3.296852 | 3.180538 |
| Glutaraldehyde | 4.2E-07 | 0.155166 | 0.007232 | 0.006531 | 0.001489 | 0.002785 | 0.005782 | 0.002895 | 0.002083 |
| Leucine | 3.504584 | 2.148353 | 2.826469 | 1.529862 | 2.15113 | 2.061129 | 2.17667 | 2.469578 | 2.208969 |
| 2-Deoxyerythritol | 0.847149 | 0.293743 | 0.570446 | 0.241771 | 0.133596 | 0.177696 | 0.112287 | 0.155473 | 0.174285 |
| N-Cyclohexylformamide | 0.071645 | 0.452284 | 0.036693 | 0.041655 | 0.038686 | 0.036353 | 0.032891 | 0.055551 | 0.044782 |
| 4-Vinylphenol | 4.2E-07 | 3.97E-06 | 1.75E-07 | 1.81E-07 | 0.002534 | 0.001043 | 0.002346 | 0.003738 | 1.61E-07 |
| Isoleucine | 4.2E-07 | 24.86808 | 1.260545 | 1.481223 | 1.271021 | 1.165798 | 1.032311 | 1.143101 | 1.132732 |
| Proline | 14.9608 | 11.03674 | 12.99877 | 13.25032 | 11.1538 | 9.970565 | 10.99703 | 11.89118 | 11.44411 |
| Phenylacetic acid | 0.024384 | 0.014063 | 0.019223 | 0.011742 | 0.011299 | 0.009811 | 0.009438 | 0.008093 | 0.009346 |
| Glycine | 4.2E-07 | 3.97E-06 | 1.75E-07 | 0.006474 | 0.005687 | 0.00547 | 0.005535 | 0.007102 | 0.006799 |
| 1,3-Cyclohexanedione | 0.002572 | 0.041008 | 0.00191 | 0.002598 | 0.002521 | 0.002193 | 0.001997 | 0.002566 | 0.00221 |
| Succinic acid | 3.785524 | 2.008453 | 2.896989 | 0.820652 | 0.738402 | 0.71918 | 1.184413 | 1.101897 | 1.090223 |
| Catechol | 0.032984 | 0.0172189 | 0.067397 | 0.020365 | 0.0196721 | 0.0219865 | 0.024216 | 0.0276321 | 0.021984 |
| 2,2-Dimethylsuccinic acid | 4.2E-07 | 0.066253 | 0.003034 | 0.002617 | 0.002627 | 0.002428 | 0.002625 | 0.003305 | 0.002598 |
| 4-Acetylbutyric acid | 0.28616 | 2.195085 | 0.118004 | 0.105438 | 0.098775 | 0.091667 | 0.091662 | 0.138687 | 0.118635 |
| D-Glyceric acid | 4.2E-07 | 1.628883 | 0.069566 | 0.065935 | 0.060834 | 0.054165 | 0.065801 | 0.076496 | 0.071824 |
| Uracil | 4.2E-07 | 25.85826 | 1.128048 | 1.110384 | 1.0319 | 0.966464 | 1.097426 | 1.189208 | 1.11289 |
| 2,3-Dimethylsuccinic acid | 4.2E-07 | 0.030363 | 1.75E-07 | 0.001071 | 1.56E-07 | 0.001081 | 0.001017 | 0.001245 | 1.61E-07 |
| Fumaric acid | 0.019914 | 0.01311 | 0.016512 | 0.008153 | 0.006552 | 0.007846 | 0.00931 | 0.010309 | 0.00853 |
| Serine | 8.371964 | 5.79526 | 7.083612 | 4.480649 | 3.043254 | 0.714173 | 4.787665 | 1.7006 | 0.846084 |
| 3-Hydroxynorvaline | 0.081978 | 1.20903 | 0.060814 | 0.183275 | 0.145091 | 0.151521 | 0.131527 | 0.15466 | 0.144746 |
| Cycloleucine | 0.19127 | 0.118486 | 0.154878 | 0.192506 | 0.179486 | 0.093467 | 0.162605 | 0.138623 | 0.166693 |
| 3-Cyanoalanine | 0.045534 | 0.614078 | 0.03334 | 1.81E-07 | 1.56E-07 | 0.052704 | 1.71E-07 | 1.74E-07 | 1.61E-07 |
| 3-Methylamino-1,2-propanediol | 0.096328 | 0.051296 | 0.073812 | 0.039865 | 0.035284 | 0.037406 | 0.041408 | 0.049415 | 0.044922 |
| L-Allothreonine | 0.979836 | 0.730879 | 0.855358 | 0.555152 | 0.353318 | 0.43002 | 0.62461 | 0.672638 | 0.647548 |
| 4-Methyl-5-thiazolethanol | 4.2E-07 | 3.97E-06 | 1.75E-07 | 0.019795 | 0.013005 | 0.016989 | 0.005339 | 0.004532 | 0.002175 |
| 4-Methylcatechol | 4.2E-07 | 3.97E-06 | 1.75E-07 | 0.000555 | 0.000698 | 1.55E-07 | 0.000732 | 0.000665 | 0.000524 |
| O-Acetylserine | 0.008784 | 0.007967 | 0.008376 | 0.019297 | 0.008491 | 0.010552 | 0.006376 | 0.015361 | 0.013756 |
| Glutaric acid | 4.2E-07 | 3.97E-06 | 1.75E-07 | 0.000905 | 1.56E-07 | 0.000752 | 0.001064 | 1.74E-07 | 0.001069 |
| Thymine | 4.2E-07 | 8.287843 | 0.344145 | 0.336521 | 0.313055 | 0.290165 | 0.333549 | 0.388411 | 0.344221 |
| DL-Anabasine | 4.2E-07 | 0.493073 | 0.022622 | 0.021903 | 0.020026 | 0.018958 | 0.019317 | 0.022827 | 0.021069 |
| Biphenyl | 4.2E-07 | 3.97E-06 | 1.75E-07 | 0.003191 | 1.56E-07 | 1.55E-07 | 0.002834 | 0.003349 | 0.003573 |
| 2-Methylglutaric acid | 0.018383 | 0.034419 | 0.002388 | 1.81E-07 | 1.56E-07 | 0.001869 | 0.002507 | 0.002514 | 0.002592 |
| Methyl trans-cinnamate | 4.2E-07 | 3.97E-06 | 0.026492 | 0.01754 | 1.56E-07 | 0.024902 | 1.71E-07 | 1.74E-07 | 0.015091 |
| N-Ethylglycine | 4.2E-07 | 3.97E-06 | 0.011431 | 0.00972 | 0.00872 | 0.009532 | 0.009047 | 0.011833 | 1.61E-07 |
| Aspartic acid | 0.009038 | 3.97E-06 | 1.75E-07 | 0.003195 | 1.56E-07 | 0.003256 | 0.003103 | 0.004084 | 1.61E-07 |
| beta-Alanine | 4.2E-07 | 4.508245 | 0.300916 | 0.191445 | 0.180642 | 0.229366 | 0.227476 | 0.258167 | 0.237729 |
| D-Erythronolactone | 4.2E-07 | 3.97E-06 | 1.75E-07 | 1.81E-07 | 0.004562 | 0.004759 | 1.71E-07 | 1.74E-07 | 0.005268 |
| Salicyl alcohol | 0.004405 | 3.97E-06 | 1.75E-07 | 0.00126 | 0.000207 | 0.000518 | 1.71E-07 | 0.001991 | 1.61E-07 |
| L-Homoserine | 4.2E-07 | 3.97E-06 | 0.08557 | 0.085569 | 0.077533 | 0.070021 | 0.078052 | 0.088492 | 0.083272 |
| Erythrose | 0.044766 | 0.470698 | 0.026899 | 0.019837 | 0.019581 | 0.026238 | 0.024431 | 0.028005 | 0.031103 |
| L-Threose | 2.23471 | 20.30331 | 0.699778 | 1.077242 | 1.12545 | 0.395372 | 1.160515 | 1.74E-07 | 1.479195 |
| Capric acid | 4.2E-07 | 3.97E-06 | 1.75E-07 | 1.81E-07 | 0.00133 | 0.001689 | 1.71E-07 | 1.74E-07 | 0.002062 |
| 3-Aminoisobutyric acid | 0.071993 | 0.568806 | 0.027237 | 0.039752 | 0.037553 | 0.02061 | 0.028622 | 0.025136 | 0.044491 |
| 2-Hydroxyacetophenone | 0.006329 | 3.97E-06 | 1.75E-07 | 0.003283 | 1.56E-07 | 0.004578 | 0.002457 | 1.74E-07 | 0.00463 |
| Aminomalonic acid | 4.2E-07 | 2.166085 | 0.108858 | 0.106335 | 0.103912 | 0.093205 | 0.099778 | 0.103487 | 0.107541 |
| (S)-Mandelic acid | 0.023276 | 3.97E-06 | 0.0179 | 0.017054 | 0.015537 | 0.014269 | 1.71E-07 | 0.02143 | 0.022633 |
| Bis(2-hydroxypropyl)amine | 0.007461 | 0.092523 | 0.003757 | 0.00151 | 0.001272 | 0.001304 | 0.003404 | 0.004063 | 1.61E-07 |
| N-Ethylmaleamic acid | 0.017542 | 0.216375 | 0.009147 | 0.008473 | 0.008935 | 0.00584 | 0.009371 | 0.010374 | 0.010368 |
| L-Malic acid | 0.010357 | 0.09306 | 0.003532 | 0.00673 | 1.56E-07 | 0.009857 | 0.005307 | 0.006802 | 0.007716 |
| 4-Hydroxycyclohexanecarboxylic acid | 4.2E-07 | 3.97E-06 | 1.75E-07 | 0.005731 | 1.56E-07 | 1.55E-07 | 0.005705 | 1.74E-07 | 0.002907 |
| 5,6-Dihydrouracil | 0.118763 | 0.961029 | 0.049909 | 0.044154 | 0.040153 | 0.040912 | 0.038862 | 1.74E-07 | 0.054418 |
| Ethyl cinnamate | 4.2E-07 | 3.97E-06 | 1.75E-07 | 0.004443 | 1.56E-07 | 0.002918 | 1.71E-07 | 1.74E-07 | 0.006415 |
| Threitol | 0.146425 | 1.972377 | 1.75E-07 | 0.08817 | 0.08308 | 0.077713 | 0.07159 | 0.081254 | 0.073779 |
| 1,5-Anhydroglucitol | 0.038837 | 3.97E-06 | 0.026198 | 1.81E-07 | 1.56E-07 | 0.012252 | 0.010647 | 1.74E-07 | 0.02174 |
| N-Acetyl-L-leucine | 4.2E-07 | 3.97E-06 | 1.75E-07 | 1.81E-07 | 0.022258 | 1.55E-07 | 0.023571 | 0.024871 | 0.02383 |
| 4-Acetamidobutyric acid | 4.2E-07 | 1.775387 | 0.140651 | 0.144626 | 0.140067 | 0.095383 | 0.111895 | 0.144844 | 0.161944 |
| Methionine | 0.035565 | 0.446413 | 0.018544 | 1.81E-07 | 0.02916 | 0.025697 | 0.01782 | 0.023621 | 0.023589 |
| Iminodiacetic acid | 6.499103 | 87.658 | 1.75E-07 | 3.316848 | 2.418943 | 2.867895 | 2.920797 | 2.889343 | 2.584095 |
| Oxoproline | 24.08174 | 79.31576 | 16.03577 | 12.72128 | 11.64615 | 11.11345 | 20.94289 | 20.73973 | 21.83847 |
| Cytosin | 4.2E-07 | 3.97E-06 | 1.75E-07 | 1.81E-07 | 1.56E-07 | 1.55E-07 | 0.146615 | 0.173684 | 0.15496 |
| Benzoylformic acid | 4.2E-07 | 3.97E-06 | 1.75E-07 | 1.81E-07 | 0.001493 | 0.001505 | 1.71E-07 | 1.74E-07 | 0.000822 |
| 4-Aminobutyric acid | 0.139619 | 0.701669 | 0.058053 | 0.032956 | 0.034398 | 0.046994 | 0.020063 | 0.048462 | 0.029293 |
| 4-Hydroxyquinazoline | 4.2E-07 | 0.245197 | 0.010583 | 1.81E-07 | 1.56E-07 | 1.55E-07 | 0.009868 | 0.01003 | 0.009247 |
| Maleamate | 4.2E-07 | 0.557924 | 0.027809 | 0.030132 | 0.026967 | 0.025232 | 0.023425 | 0.028123 | 0.026771 |
| Pyrogallol | 4.2E-07 | 3.97E-06 | 1.75E-07 | 1.81E-07 | 1.56E-07 | 1.55E-07 | 0.006992 | 0.00981 | 0.008098 |
| Nornicotine | 4.2E-07 | 0.385056 | 0.02191 | 0.019999 | 0.017991 | 0.017446 | 0.01794 | 0.019804 | 0.02012 |
| Malonamide | 4.2E-07 | 0.730204 | 0.034627 | 0.031283 | 0.028052 | 0.026064 | 0.028033 | 0.032005 | 0.029902 |
| Threonic acid | 0.557467 | 0.426731 | 0.492099 | 0.612924 | 0.55217 | 0.52482 | 0.446483 | 0.490506 | 0.453121 |
| Creatine | 4.2E-07 | 3.97E-06 | 1.75E-07 | 1.81E-07 | 1.56E-07 | 1.55E-07 | 0.015727 | 0.018934 | 0.030305 |
| Phenylethylamine | 0.181066 | 0.177451 | 0.179259 | 0.154329 | 0.14027 | 0.164946 | 0.08863 | 0.105657 | 0.108013 |
| 2-Hydroxy-3-isopropylbutanedioic acid | 0.463418 | 7.231001 | 0.358437 | 0.762231 | 0.677524 | 0.708448 | 0.542521 | 0.611375 | 0.574114 |
| (2R,3S)-2-Hydroxy-3-isopropylbutanedioic acid | 4.2E-07 | 0.246196 | 1.75E-07 | 0.018929 | 0.017336 | 0.01717 | 0.017224 | 0.019455 | 0.017291 |
| threo-beta-Hyrdoxyaspartate | 0.334319 | 4.224019 | 0.231224 | 0.095592 | 0.065603 | 0.16798 | 0.136911 | 0.166233 | 0.169946 |
| 3-Phenyllactic acid | 0.25789 | 0.734284 | 0.323104 | 0.728252 | 0.736473 | 0.654603 | 0.81739 | 0.933053 | 0.875221 |
| Phosphoglycolic acid | 0.007213 | 0.086673 | 0.004242 | 0.002896 | 0.002483 | 0.002354 | 0.005133 | 0.005293 | 0.005083 |
| 3-Hydroxy-3-methylglutaric acid | 4.2E-07 | 1.672206 | 0.072623 | 0.070835 | 0.058776 | 0.066894 | 0.070574 | 0.077949 | 0.07149 |
| Hexadecane | 4.2E-07 | 3.97E-06 | 1.75E-07 | 1.81E-07 | 0.045727 | 1.55E-07 | 1.71E-07 | 0.080494 | 0.070915 |
| Digitoxose | 3.232008 | 1.231722 | 2.231865 | 0.660283 | 0.502526 | 0.38243 | 0.767442 | 0.853057 | 0.821279 |
| 3-Hydroxyphenylacetic acid | 4.2E-07 | 0.066722 | 1.75E-07 | 1.81E-07 | 1.56E-07 | 1.55E-07 | 1.71E-07 | 0.001392 | 0.00091 |
| Glutamic acid | 0.227433 | 0.204174 | 0.215804 | 0.171703 | 0.145584 | 0.145889 | 0.700864 | 0.813228 | 0.868254 |
| Phenylalanine | 4.2E-07 | 3.97E-06 | 1.75E-07 | 1.81E-07 | 1.56E-07 | 0.041807 | 0.054654 | 0.049975 | 0.055975 |
| 4-Hydroxybenzoic acid | 4.2E-07 | 3.349334 | 1.75E-07 | 0.146222 | 0.130637 | 0.128781 | 0.136938 | 0.153462 | 0.1452 |
| Creatine degr | 4.2E-07 | 3.97E-06 | 0.055244 | 0.047826 | 0.042734 | 0.055576 | 0.043667 | 0.054818 | 0.06305 |
| Tartaric acid | 4.2E-07 | 3.97E-06 | 1.75E-07 | 1.81E-07 | 0.01037 | 0.009604 | 0.006755 | 0.007367 | 0.007167 |
| 5-Aminovaleric acid | 4.2E-07 | 0.823685 | 0.055218 | 0.064821 | 0.050761 | 0.073311 | 0.047304 | 0.064926 | 0.063402 |
| 4-Hydroxy-3-methoxybenzyl alcohol | 4.2E-07 | 3.97E-06 | 1.75E-07 | 0.001247 | 1.56E-07 | 0.00125 | 1.71E-07 | 0.000964 | 1.61E-07 |
| 4-Hydroxyphenylacetic acid | 0.063293 | 3.97E-06 | 1.75E-07 | 0.026887 | 0.022749 | 0.022571 | 1.71E-07 | 1.74E-07 | 0.023915 |
| Lyxose | 10.28809 | 3.339824 | 6.81396 | 0.909291 | 0.172953 | 0.330554 | 0.474484 | 0.818519 | 0.486831 |
| 1,3-Diaminopropane | 4.2E-07 | 5.983112 | 0.403607 | 0.373133 | 0.310994 | 0.288082 | 0.335964 | 0.384597 | 0.37085 |
| Allose | 4.2E-07 | 3.97E-06 | 1.75E-07 | 0.011753 | 0.001396 | 1.55E-07 | 0.00937 | 1.74E-07 | 0.003756 |
| Lauric acid | 4.2E-07 | 3.97E-06 | 1.75E-07 | 0.030905 | 0.012387 | 0.018418 | 0.0306 | 0.021652 | 0.026126 |
| Xylose | 0.466821 | 7.184397 | 1.75E-07 | 0.482733 | 0.009354 | 0.018509 | 0.097663 | 0.139968 | 0.026507 |
| Asparagine | 0.049707 | 0.064351 | 0.057029 | 0.036017 | 0.004302 | 0.015663 | 0.036371 | 0.040915 | 0.044947 |
| Ribonic acid, gamma-lactone | 0.547727 | 0.3809 | 0.464313 | 0.285854 | 0.119319 | 0.330313 | 0.266951 | 0.401007 | 0.326523 |
| Ribose | 4.2E-07 | 0.710201 | 0.029401 | 0.020135 | 0.012082 | 0.028759 | 0.01278 | 0.01455 | 0.009921 |
| 3-Ureidopropionate | 4.2E-07 | 3.97E-06 | 1.75E-07 | 1.81E-07 | 1.56E-07 | 0.010165 | 0.012911 | 0.015444 | 0.016073 |
| Xylitol | 1.943027 | 0.462608 | 1.202818 | 1.890498 | 1.267972 | 1.340568 | 0.651455 | 0.741443 | 0.771254 |
| Biuret | 4.2E-07 | 3.058408 | 0.175231 | 1.81E-07 | 0.089575 | 0.107068 | 0.109138 | 0.143852 | 0.122937 |
| 6-deoxy-D-Glucose | 4.2E-07 | 3.97E-06 | 1.75E-07 | 0.08602 | 0.081613 | 0.085994 | 1.71E-07 | 1.74E-07 | 1.61E-07 |
| Fucose | 0.785362 | 0.15071 | 0.468036 | 0.5758 | 0.494521 | 0.535161 | 0.576343 | 0.407437 | 0.629013 |
| Ribitol | 4.2E-07 | 3.97E-06 | 0.067238 | 0.062734 | 0.056298 | 0.055547 | 0.063551 | 0.068092 | 0.062862 |
| Putrescine | 0.037723 | 0.010472 | 0.024098 | 0.037302 | 0.03732 | 0.037311 | 0.042335 | 0.042729 | 0.042598 |
| Glutaconic acid | 4.2E-07 | 3.97E-06 | 0.138565 | 0.000349 | 1.56E-07 | 1.55E-07 | 1.71E-07 | 0.12843 | 0.0005 |
| Lyxonic acid, 1,4-lactone | 4.2E-07 | 3.97E-06 | 0.003077 | 1.81E-07 | 1.56E-07 | 0.003156 | 0.002953 | 0.003184 | 1.61E-07 |
| Orotic acid | 0.017245 | 0.1432 | 0.008692 | 1.81E-07 | 1.56E-07 | 1.55E-07 | 1.71E-07 | 0.008759 | 0.008598 |
| Carbobenzyloxy-L-leucine degr3 | 0.025208 | 3.97E-06 | 1.75E-07 | 0.020832 | 0.018539 | 1.55E-07 | 0.026652 | 1.74E-07 | 0.026041 |
| Hexachlorobenzene | 0.173063 | 3.1262 | 1.75E-07 | 0.092387 | 0.073382 | 0.085118 | 0.124372 | 1.74E-07 | 0.140396 |
| flavin adenine degrad product | 4.2E-07 | 0.310345 | 0.015101 | 0.011626 | 0.011301 | 0.011463 | 0.016216 | 0.012626 | 0.011354 |
| Aconitic acid | 4.2E-07 | 1.393649 | 0.035171 | 0.011689 | 0.007646 | 0.013832 | 0.016753 | 0.009897 | 0.00786 |
| Diglycerol | 0.093765 | 0.005185 | 0.049475 | 0.006175 | 0.005408 | 0.005602 | 0.004841 | 0.00424 | 0.004024 |
| D-(glycerol 1-phosphate) | 0.009286 | 0.005928 | 0.007607 | 0.005042 | 0.003979 | 0.005074 | 0.005518 | 0.00479 | 0.005154 |
| Glucose-1-phosphate | 4.2E-07 | 3.926377 | 0.172297 | 0.250163 | 0.186533 | 0.218962 | 0.163091 | 0.207458 | 0.196857 |
| 3,6-Anhydro-D-galactose | 0.016158 | 0.271868 | 0.012652 | 0.015166 | 0.014209 | 0.013033 | 1.71E-07 | 0.010722 | 0.00996 |
| 5,6-Dimethylbenzimidazole | 4.2E-07 | 25.70412 | 1.017111 | 1.329469 | 1.02793 | 1.20041 | 1.247988 | 1.233507 | 1.248307 |
| 3-(4-hydroxyphenyl)Propionic acid | 4.2E-07 | 67.4305 | 2.613799 | 3.486958 | 3.406691 | 3.259737 | 3.425191 | 3.454815 | 3.474008 |
| 4-Hydroxy-3-methoxybenzoic acid | 4.2E-07 | 3.962936 | 0.149652 | 0.159072 | 0.142146 | 0.140777 | 0.151996 | 0.166268 | 0.151724 |
| Gentisic acid | 4.2E-07 | 2.135597 | 0.099161 | 0.096116 | 0.088754 | 0.089889 | 0.096439 | 0.104729 | 0.097317 |
| N-Acetyl-L-glutamic acid | 0.247068 | 5.47167 | 0.203998 | 0.132954 | 0.086623 | 0.122139 | 0.253643 | 0.265981 | 0.262028 |
| Methionine sulfoxide | 0.026239 | 0.394399 | 0.016451 | 0.024821 | 1.56E-07 | 1.55E-07 | 0.039384 | 0.047034 | 1.61E-07 |
| 5-Aminoimidazole-4-carboxamide | 4.2E-07 | 0.759679 | 0.040076 | 0.036205 | 0.037018 | 0.033318 | 0.038565 | 0.036645 | 0.038332 |
| 2-deoxy-D-Glucose | 0.009581 | 3.97E-06 | 1.75E-07 | 0.005181 | 1.56E-07 | 0.004868 | 1.71E-07 | 0.005707 | 1.61E-07 |
| 2-Deoxy-D-Galactose | 4.2E-07 | 12.37408 | 0.56351 | 0.573147 | 0.50739 | 0.512879 | 0.674509 | 0.687495 | 0.65954 |
| Methoxamedrine | 4.2E-07 | 3.97E-06 | 1.75E-07 | 0.0055 | 0.005158 | 0.005271 | 0.006395 | 0.00649 | 0.005679 |
| Azelaic acid | 4.2E-07 | 0.575074 | 0.019899 | 0.02141 | 0.019361 | 0.020039 | 0.022816 | 0.024265 | 0.02117 |
| Cysteinylglycine | 0.042002 | 0.756827 | 1.75E-07 | 0.035574 | 0.03063 | 0.034561 | 0.050749 | 0.053456 | 0.047959 |
| Shikimic acid | 4.2E-07 | 3.97E-06 | 1.75E-07 | 0.000916 | 1.56E-07 | 1.55E-07 | 0.000437 | 0.000695 | 1.61E-07 |
| Hypoxanthine | 4.2E-07 | 2.753742 | 0.118423 | 0.101048 | 1.56E-07 | 1.55E-07 | 0.145694 | 1.74E-07 | 0.140955 |
| Ornithin | 4.2E-07 | 52.73672 | 1.75E-07 | 3.197355 | 2.569552 | 2.304864 | 2.364591 | 2.832371 | 3.018311 |
| alpha-D-glucosamine 1-phosphate | 4.2E-07 | 3.97E-06 | 1.75E-07 | 0.42257 | 0.378874 | 0.282807 | 1.71E-07 | 1.74E-07 | 1.61E-07 |
| Isocitric acid | 4.2E-07 | 3.97E-06 | 1.75E-07 | 1.81E-07 | 1.56E-07 | 0.087699 | 0.135875 | 0.090767 | 0.103909 |
| 3,4-Dihydroxybenzoic acid | 0.541877 | 0.27559 | 0.408733 | 0.258553 | 0.244925 | 0.236816 | 0.083067 | 0.089189 | 0.082411 |
| N-Acetylisatin | 4.2E-07 | 3.97E-06 | 1.75E-07 | 1.81E-07 | 1.56E-07 | 0.014473 | 0.019806 | 0.015529 | 0.018892 |
| Synephrine | 4.2E-07 | 3.97E-06 | 1.75E-07 | 0.017383 | 0.016054 | 1.55E-07 | 0.016299 | 1.74E-07 | 1.61E-07 |
| Myristic acid | 0.058831 | 0.024406 | 0.041619 | 0.027482 | 0.01978 | 0.02442 | 0.024594 | 0.031279 | 0.027937 |
| Guanidinosuccinic acid | 4.2E-07 | 3.97E-06 | 0.015632 | 0.014995 | 0.013509 | 0.012699 | 0.016347 | 0.014926 | 0.015944 |
| Quinic acid | 0.012786 | 0.00918 | 0.010983 | 0.008047 | 0.006385 | 0.006615 | 1.71E-07 | 1.74E-07 | 1.61E-07 |
| Tagatose | 1.168846 | 3.183799 | 0.921086 | 1.290513 | 1.111313 | 1.159718 | 1.502727 | 1.561266 | 1.418022 |
| Allo-inositol | 4.2E-07 | 3.97E-06 | 1.75E-07 | 0.011516 | 0.001993 | 0.002732 | 0.001121 | 1.74E-07 | 0.011074 |
| Sorbose | 4.2E-07 | 3.97E-06 | 1.75E-07 | 0.002596 | 0.002258 | 1.55E-07 | 0.003244 | 0.002992 | 1.61E-07 |
| Fructose | 0.046902 | 3.97E-06 | 1.75E-07 | 0.033564 | 0.004959 | 1.55E-07 | 0.007097 | 0.016011 | 1.61E-07 |
| Adenine | 4.2E-07 | 14.4188 | 0.743247 | 0.837105 | 0.710425 | 0.750357 | 0.676947 | 0.724805 | 0.658404 |
| Adipamide | 0.227466 | 1.586113 | 0.049933 | 1.81E-07 | 1.56E-07 | 1.55E-07 | 0.181913 | 0.203134 | 0.199451 |
| Vanillylmandelic acid | 4.2E-07 | 3.97E-06 | 1.75E-07 | 0.194086 | 0.150249 | 1.55E-07 | 0.218134 | 0.236549 | 0.254099 |
| Gluconic lactone | 4.2E-07 | 3.97E-06 | 1.75E-07 | 0.005542 | 0.004951 | 0.005246 | 0.004769 | 0.004302 | 0.004536 |
| Mannose | 4.2E-07 | 3.97E-06 | 1.75E-07 | 0.094148 | 0.039331 | 0.105459 | 0.000274 | 1.74E-07 | 1.61E-07 |
| D-Altrose | 0.151375 | 3.97E-06 | 0.06247 | 0.075071 | 1.56E-07 | 0.059646 | 0.063864 | 0.078141 | 0.0624 |
| 2-Keto-L-Gulonic acid | 4.2E-07 | 3.97E-06 | 1.75E-07 | 0.059687 | 0.047348 | 0.056187 | 1.71E-07 | 1.74E-07 | 1.61E-07 |
| Glucose | 0.012784 | 0.031199 | 0.00454 | 0.013225 | 0.010656 | 0.014934 | 0.005893 | 0.008521 | 0.012183 |
| D-Talose | 4.2E-07 | 25.83316 | 0.422773 | 0.085321 | 0.075717 | 0.061511 | 1.121542 | 1.52889 | 0.351269 |
| dl-p-Hydroxyphenyllactic acid | 0.215905 | 3.97E-06 | 1.75E-07 | 0.089011 | 0.067685 | 0.065716 | 0.088714 | 0.086661 | 0.087227 |
| Galactose | 4.2E-07 | 3.97E-06 | 0.796652 | 2.817346 | 1.56E-07 | 1.701998 | 1.71E-07 | 0.239912 | 1.61E-07 |
| Methyl Palmitoleate | 0.22439 | 1.541265 | 0.087764 | 0.073652 | 0.08079 | 0.064418 | 0.06925 | 0.084827 | 0.088026 |
| Tyramine | 4.2E-07 | 136.094 | 1.75E-07 | 6.186373 | 5.236443 | 5.253563 | 5.339693 | 5.731484 | 5.498407 |
| Lysine | 4.2E-07 | 3.97E-06 | 1.75E-07 | 0.974269 | 1.56E-07 | 1.55E-07 | 0.663909 | 0.817398 | 0.740654 |
| Mannitol | 0.031923 | 0.707613 | 0.026464 | 0.091488 | 0.100043 | 0.029749 | 0.10128 | 0.108374 | 0.095312 |
| Sorbitol | 4.2E-07 | 3.97E-06 | 0.006306 | 0.008572 | 0.002886 | 0.003766 | 0.005798 | 0.004866 | 0.005332 |
| D-Galacturonic acid | 0.202401 | 3.97E-06 | 1.75E-07 | 0.053139 | 0.043633 | 0.061835 | 0.121702 | 0.185478 | 0.237176 |
| Tyrosine | 4.2E-07 | 3.97E-06 | 1.75E-07 | 0.07794 | 0.101301 | 0.082661 | 0.201048 | 0.277762 | 0.172917 |
| Coniferyl alcohol | 4.2E-07 | 3.97E-06 | 1.75E-07 | 0.007398 | 1.56E-07 | 0.007506 | 1.71E-07 | 0.010801 | 0.008337 |
| 4-Hydroxycinnamic acid | 4.2E-07 | 3.97E-06 | 1.75E-07 | 0.327052 | 0.295082 | 0.27522 | 0.317604 | 0.327823 | 0.322713 |
| Pentadecanoic acid | 4.2E-07 | 3.97E-06 | 1.75E-07 | 0.005301 | 0.004099 | 0.0047 | 0.004551 | 0.005283 | 0.004426 |
| Conduritol-β-epoxide | 4.2E-07 | 3.97E-06 | 1.75E-07 | 0.018022 | 0.01855 | 0.010163 | 0.00927 | 0.011716 | 0.010493 |
| Gallic acid | 4.2E-07 | 3.97E-06 | 1.75E-07 | 0.012726 | 1.56E-07 | 0.011387 | 1.71E-07 | 0.006769 | 0.005034 |
| Sinapyl alcohol | 0.04807 | 0.017965 | 0.033017 | 0.028689 | 0.025333 | 0.024905 | 0.029985 | 0.032971 | 0.030591 |
| 4-Hydroxy-3-methoxycinnamaldehyde | 4.2E-07 | 3.97E-06 | 1.75E-07 | 1.81E-07 | 0.000246 | 1.55E-07 | 1.71E-07 | 0.02365 | 0.023134 |
| 3,5-Dihydroxyphenylglycine | 4.2E-07 | 3.97E-06 | 1.75E-07 | 0.009299 | 1.56E-07 | 0.007763 | 0.008488 | 1.74E-07 | 0.008576 |
| indole-3-Acetic acid | 4.2E-07 | 3.97E-06 | 1.75E-07 | 0.02651 | 1.56E-07 | 0.024709 | 1.71E-07 | 1.74E-07 | 0.031575 |
| Canavanine | 4.2E-07 | 3.97E-06 | 1.75E-07 | 0.002233 | 0.001681 | 0.002161 | 1.71E-07 | 1.74E-07 | 0.001224 |
| 2,6-Diaminopimelic acid | 4.2E-07 | 3.97E-06 | 1.75E-07 | 0.001843 | 0.001389 | 0.001496 | 0.006524 | 0.005007 | 0.00392 |
| Lipoic acid | 4.2E-07 | 3.97E-06 | 1.75E-07 | 0.001043 | 1.56E-07 | 0.001071 | 1.71E-07 | 0.00212 | 0.001824 |
| Gly-pro | 0.006912 | 3.97E-06 | 1.75E-07 | 0.004843 | 1.56E-07 | 1.55E-07 | 1.71E-07 | 0.005907 | 0.005298 |
| N-alpha-Acetyl-L-ornithine | 0.055815 | 3.97E-06 | 1.75E-07 | 0.017941 | 0.016702 | 1.55E-07 | 0.044213 | 0.03984 | 0.044107 |
| Galactonic acid | 0.0126 | 0.009086 | 0.010843 | 0.006689 | 0.004273 | 0.005095 | 0.003976 | 0.004133 | 0.003804 |
| Gluconic acid | 0.037768 | 3.97E-06 | 0.031948 | 0.024306 | 1.56E-07 | 0.011318 | 1.71E-07 | 0.027351 | 0.024791 |
| Saccharic acid | 0.074463 | 3.97E-06 | 1.75E-07 | 0.233207 | 0.200806 | 0.217007 | 1.71E-07 | 1.74E-07 | 0.106288 |
| Xanthine | 0.29566 | 5.963526 | 1.75E-07 | 0.226396 | 0.027555 | 0.220655 | 0.109165 | 0.038671 | 0.208509 |
| Glucosaminic acid | 4.2E-07 | 3.97E-06 | 1.75E-07 | 0.037969 | 0.034838 | 0.034355 | 0.038141 | 0.03228 | 0.035475 |
| Palmitoleic acid | 0.011193 | 0.008368 | 0.009781 | 0.005188 | 0.006023 | 0.004669 | 0.007802 | 0.008276 | 0.008188 |
| 4-Hydroxymethyl-3-methoxyphenoxyacetic acid | 4.2E-07 | 1.488177 | 0.059251 | 0.064341 | 0.059099 | 0.055482 | 0.069443 | 0.077197 | 0.06836 |
| N-Carbamylglutamate | 0.039328 | 3.97E-06 | 1.75E-07 | 1.81E-07 | 1.56E-07 | 0.023117 | 0.036065 | 1.74E-07 | 0.037038 |
| Mucic acid | 0.120988 | 3.97E-06 | 0.081564 | 1.81E-07 | 1.56E-07 | 0.035352 | 0.057853 | 0.061298 | 0.058144 |
| Palmitic acid | 10.44734 | 96.25009 | 3.920282 | 4.074247 | 3.700711 | 3.714374 | 3.613906 | 4.173097 | 3.571141 |
| Isopropyl-beta-D-thiogalactopyranoside | 4.2E-07 | 3.97E-06 | 1.75E-07 | 0.002481 | 0.001945 | 0.00211 | 0.002432 | 1.74E-07 | 0.002123 |
| N,N-Dimethylarginine | 4.2E-07 | 0.577168 | 0.031843 | 0.015405 | 0.034814 | 0.010299 | 0.015559 | 0.00607 | 0.010771 |
| N-Acetyl-D-galactosamine | 0.105933 | 2.281927 | 0.064712 | 0.013795 | 0.002663 | 0.018464 | 0.099854 | 0.117523 | 0.052009 |
| myo-Inositol | 0.680961 | 3.97E-06 | 1.75E-07 | 1.464716 | 0.259025 | 0.942764 | 0.294146 | 0.282967 | 0.345263 |
| Linoleic acid methyl ester | 1.608041 | 3.97E-06 | 0.531549 | 0.678113 | 0.740843 | 0.622391 | 0.782491 | 0.828484 | 0.805487 |
| Ferulic acid | 0.258219 | 0.43178 | 0.665408 | 0.27693 | 0.138168 | 0.17274 | 0.189965 | 0.329342 | 0.206221 |
| N-Acetyl-beta-D-mannosamine | 1.034788 | 11.35423 | 0.35776 | 0.188838 | 0.110852 | 0.013855 | 0.177189 | 0.127032 | 0.064012 |
| indole-3-Acetamide | 0.062835 | 0.45149 | 1.75E-07 | 0.018155 | 0.009806 | 1.55E-07 | 1.71E-07 | 0.023222 | 1.61E-07 |
| d-Glucoheptose | 0.04506 | 0.033273 | 0.039167 | 0.02205 | 0.020363 | 0.021206 | 0.028459 | 0.024809 | 0.0236 |
| trans-3,5-Dimethoxy-4-hydroxycinnamaldehyde | 4.2E-07 | 3.97E-06 | 1.75E-07 | 0.002603 | 0.001854 | 0.001957 | 0.003586 | 0.00364 | 1.61E-07 |
| Guanine | 0.17835 | 3.97E-06 | 1.75E-07 | 1.81E-07 | 0.059795 | 0.066087 | 0.063133 | 0.067197 | 0.051761 |
| Glucoheptonic acid | 0.216441 | 0.106926 | 0.161684 | 0.071004 | 0.026774 | 0.028592 | 0.018267 | 0.013535 | 0.015901 |
| Flavanone | 4.2E-07 | 3.97E-06 | 1.75E-07 | 0.005407 | 0.004826 | 0.004757 | 0.005622 | 0.0058 | 0.005543 |
| 3,4-Dihydroxycinnamic acid | 4.2E-07 | 3.97E-06 | 1.75E-07 | 0.034024 | 0.021629 | 0.027826 | 0.029315 | 0.034244 | 0.022085 |
| Caffeic acid | 4.2E-07 | 3.97E-06 | 1.75E-07 | 1.81E-07 | 0.002726 | 1.55E-07 | 0.004121 | 0.004137 | 1.61E-07 |
| cis-Phytol | 4.2E-07 | 3.97E-06 | 1.75E-07 | 0.006545 | 0.004817 | 1.55E-07 | 0.006941 | 0.008244 | 1.61E-07 |
| Heptadecanoic acid | 0.071015 | 0.026788 | 0.048901 | 0.027332 | 0.021014 | 0.02195 | 0.025575 | 0.028334 | 0.022743 |
| 6-Hydroxy caproic acid dimer | 0.004726 | 0.050042 | 0.001471 | 0.002504 | 0.002487 | 0.001496 | 0.002028 | 0.001306 | 0.001384 |
| Indolelactate | 0.015992 | 0.011374 | 0.013683 | 1.81E-07 | 1.56E-07 | 1.55E-07 | 0.015349 | 0.01133 | 0.013339 |
| Glutathione | 2.040606 | 21.03734 | 0.658365 | 0.686023 | 0.422637 | 0.656272 | 0.808353 | 0.928473 | 0.666906 |
| Phytol | 1.298303 | 14.45881 | 0.451351 | 0.533632 | 0.381355 | 0.44943 | 0.556857 | 0.508709 | 0.390682 |
| L-Kynurenine | 4.2E-07 | 3.97E-06 | 0.012578 | 1.81E-07 | 0.013679 | 1.55E-07 | 0.020037 | 1.74E-07 | 0.017241 |
| 3-Hydroxypalmitic acid | 4.2E-07 | 2.58152 | 0.093845 | 0.129271 | 0.085664 | 0.115952 | 0.111212 | 0.138609 | 0.110261 |
| beta-Mannosylglycerate | 0.672376 | 0.301389 | 0.486882 | 0.085957 | 0.036959 | 0.068774 | 0.177706 | 0.206968 | 0.192337 |
| Linoleic acid | 0.324057 | 2.911586 | 0.108624 | 0.08869 | 0.071308 | 0.071999 | 0.091728 | 0.11296 | 0.088907 |
| Oleic acid | 4.2E-07 | 1.661756 | 0.063614 | 1.81E-07 | 0.050497 | 0.056955 | 0.069612 | 0.082548 | 0.069385 |
| Linolenic acid | 4.2E-07 | 3.97E-06 | 0.574547 | 0.482581 | 0.34807 | 0.393091 | 0.539017 | 0.550571 | 0.446497 |
| 2-Aminoethanethiol | 4.640732 | 2.986275 | 3.813504 | 3.62473 | 3.056715 | 2.881672 | 2.190268 | 2.294416 | 1.158148 |
| trans-Sinapinic acid | 0.320456 | 0.012752 | 0.166604 | 0.010565 | 0.002052 | 0.006308 | 0.013929 | 0.01474 | 0.013865 |
| Stearic acid | 2.277566 | 20.78798 | 0.846162 | 0.862988 | 0.727989 | 0.760893 | 0.923132 | 1.74E-07 | 1.61E-07 |
| Pyridoxal phosphate | 0.008979 | 0.365221 | 0.012705 | 1.81E-07 | 1.56E-07 | 1.55E-07 | 1.71E-07 | 1.74E-07 | 1.61E-07 |
| Xanthurenic acid | 4.2E-07 | 0.113453 | 1.75E-07 | 0.004996 | 0.005182 | 1.55E-07 | 0.005438 | 0.006196 | 0.005182 |
| spermidine | 0.006843 | 0.105888 | 0.003571 | 0.004899 | 0.00339 | 0.00403 | 0.004748 | 0.004731 | 1.61E-07 |
| alpha-Santonin | 4.2E-07 | 3.97E-06 | 0.006784 | 0.005499 | 0.004945 | 0.005564 | 1.71E-07 | 1.74E-07 | 1.61E-07 |
| Fructose-6-phosphate | 0.002758 | 3.97E-06 | 1.75E-07 | 0.001004 | 0.001263 | 0.001161 | 0.001739 | 0.001359 | 0.001227 |
| Atropine | 0.130808 | 1.200484 | 0.060592 | 0.060747 | 0.063455 | 0.046627 | 0.046683 | 0.05419 | 0.062589 |
| Phenyl beta-D-glucopyranoside | 4.2E-07 | 0.1693 | 1.75E-07 | 0.008154 | 0.006103 | 0.005742 | 0.007524 | 1.74E-07 | 1.61E-07 |
| Glucose-6-phosphate | 0.011448 | 3.97E-06 | 0.008788 | 0.009968 | 0.009102 | 0.008942 | 1.71E-07 | 0.017671 | 0.014433 |
| 3-Hydroxyflavone | 4.2E-07 | 0.174139 | 0.007464 | 0.010533 | 0.008228 | 0.009328 | 0.011485 | 0.009482 | 0.008415 |
| Purine riboside | 0.021992 | 0.013044 | 0.017518 | 1.81E-07 | 1.56E-07 | 1.55E-07 | 0.010733 | 0.013436 | 0.011758 |
| Arachidonic acid | 0.009426 | 0.008525 | 0.009157 | 0.00858 | 0.009597 | 0.009089 | 0.007743 | 0.008329 | 0.007655 |
| 1-Methyladenosine | 4.2E-07 | 0.588012 | 0.03214 | 0.033096 | 1.56E-07 | 0.006237 | 0.045972 | 0.054029 | 1.61E-07 |
| Abietic acid | 4.2E-07 | 0.330083 | 0.011626 | 1.81E-07 | 0.010938 | 1.55E-07 | 1.71E-07 | 1.74E-07 | 1.61E-07 |
| cis-Gondoic acid | 4.2E-07 | 0.075103 | 0.000185 | 0.002498 | 0.003 | 0.002749 | 0.003794 | 0.001293 | 0.002543 |
| 6-Phosphogluconic acid | 0.337698 | 2.984183 | 0.127491 | 1.81E-07 | 1.56E-07 | 1.55E-07 | 0.086905 | 0.07445 | 0.055744 |
| Arachidic acid | 0.267664 | 0.111017 | 0.189341 | 0.121117 | 0.099263 | 0.105074 | 0.10916 | 0.1161 | 0.110088 |
| D-erythro-sphingosine | 4.2E-07 | 3.97E-06 | 1.75E-07 | 1.81E-07 | 1.56E-07 | 0.000709 | 1.71E-07 | 0.003401 | 0.002985 |
| 4-Vinylphenol dimer | 4.2E-07 | 3.97E-06 | 0.001624 | 0.003276 | 0.004228 | 0.003377 | 1.71E-07 | 1.74E-07 | 1.61E-07 |
| Uridine | 0.014289 | 0.009215 | 0.011752 | 0.001391 | 0.008233 | 0.004812 | 0.004774 | 0.006308 | 0.004621 |
| 5-Methoxytryptamine | 4.2E-07 | 3.97E-06 | 0.011413 | 0.004372 | 1.56E-07 | 0.007753 | 1.71E-07 | 0.013274 | 1.61E-07 |
| Neohesperidin | 0.040887 | 0.003364 | 0.022125 | 0.002221 | 0.0025 | 0.001197 | 0.002306 | 0.003749 | 0.003027 |
| Salicin | 4.2E-07 | 3.97E-06 | 0.014316 | 0.014601 | 0.011572 | 0.010017 | 0.016542 | 0.016717 | 0.015716 |
| Homocystine | 0.054793 | 0.095263 | 0.075028 | 0.084257 | 0.065638 | 0.050557 | 0.02595 | 0.053457 | 0.039703 |
| 2-Monopalmitin | 0.013518 | 0.307552 | 0.009575 | 0.015527 | 0.007299 | 0.004063 | 0.003203 | 0.016617 | 0.00991 |
| Arbutin | 0.008345 | 3.97E-06 | 1.75E-07 | 0.004051 | 0.003317 | 1.55E-07 | 1.71E-07 | 1.74E-07 | 1.61E-07 |
| Androsterone | 4.2E-07 | 0.212526 | 1.75E-07 | 0.012165 | 0.008186 | 0.010175 | 0.016373 | 0.012433 | 0.01246 |
| kyotorphin | 0.162939 | 0.081627 | 0.122283 | 0.091624 | 0.076995 | 0.08431 | 0.097206 | 0.093154 | 0.091337 |
| 1-Monopalmitin | 0.126742 | 0.04587 | 0.086306 | 0.066091 | 0.046515 | 0.065905 | 0.039516 | 0.078304 | 0.070582 |
| Phytosphingosine | 0.00643 | 0.002761 | 0.004595 | 1.81E-07 | 0.002432 | 1.55E-07 | 0.002469 | 0.002785 | 0.002627 |
| Prostaglandin | 0.013772 | 0.159483 | 0.004929 | 0.005595 | 1.56E-07 | 0.005614 | 1.71E-07 | 0.006047 | 0.004755 |
| Sucrose | 0.023566 | 3.97E-06 | 1.75E-07 | 0.007 | 0.008686 | 1.55E-07 | 1.71E-07 | 1.74E-07 | 0.007504 |
| Behenic acid | 0.120384 | 0.042545 | 0.081465 | 0.051676 | 0.037395 | 0.039898 | 0.044941 | 0.047076 | 0.042448 |
| Androstanediol | 0.000847 | 0.001704 | 0.001276 | 0.001968 | 0.001069 | 0.002228 | 1.71E-07 | 1.74E-07 | 0.001163 |
| Lactose | 0.35048 | 0.229194 | 0.289837 | 0.064943 | 0.052251 | 0.049354 | 0.054114 | 0.056975 | 0.045848 |
| Dihydrotestosterone | 4.2E-07 | 0.035466 | 0.001026 | 1.81E-07 | 1.56E-07 | 1.55E-07 | 1.71E-07 | 0.000383 | 1.61E-07 |
| Lactulose | 0.004935 | 0.004373 | 0.004654 | 0.001458 | 0.004013 | 0.002735 | 0.003844 | 0.005793 | 0.001818 |
| 11-beta-prostaglandin-F-2-alpha | 4.2E-07 | 0.844074 | 1.75E-07 | 1.81E-07 | 0.040893 | 1.55E-07 | 0.037658 | 1.74E-07 | 0.028552 |
| Cellobiose | 4.2E-07 | 3.97E-06 | 1.75E-07 | 0.034259 | 0.027241 | 0.03075 | 0.040669 | 0.036817 | 0.038743 |
| Trehalose | 0.105519 | 3.97E-06 | 0.139115 | 0.066067 | 1.56E-07 | 1.55E-07 | 0.087991 | 0.070172 | 0.06309 |
| Monoolein | 0.000364 | 0.004879 | 1.75E-07 | 0.000478 | 0.000491 | 0.000485 | 1.71E-07 | 1.74E-07 | 6.7E-05 |
| Sophorose | 4.2E-07 | 3.97E-06 | 0.016462 | 0.008555 | 0.005745 | 0.007371 | 0.011182 | 0.014305 | 0.017536 |
| Leucrose | 4.2E-07 | 0.395371 | 0.015886 | 0.001638 | 0.005512 | 0.001411 | 0.005939 | 0.002402 | 0.00417 |
| Monostearin | 0.055509 | 0.021712 | 0.03861 | 0.03154 | 0.031669 | 0.031605 | 0.019721 | 0.035037 | 0.034804 |
| Gentiobiose | 4.2E-07 | 0.031275 | 0.002289 | 0.002389 | 0.001416 | 0.001495 | 0.000868 | 0.001899 | 0.001384 |
| Prostaglandin | 0.001271 | 0.01699 | 1.75E-07 | 1.81E-07 | 1.56E-07 | 0.000294 | 1.71E-07 | 1.74E-07 | 1.61E-07 |
| Squalene | 4.2E-07 | 1.452862 | 0.057638 | 0.067666 | 0.051257 | 0.058278 | 0.067152 | 0.060144 | 0.063648 |
| prunin degr. Prod. | 4.2E-07 | 3.97E-06 | 0.032131 | 1.81E-07 | 0.027505 | 0.021198 | 1.71E-07 | 0.024034 | 1.61E-07 |
| Lignoceric acid | 4.2E-07 | 1.572742 | 0.062283 | 0.069098 | 0.048587 | 0.056527 | 0.06651 | 0.060707 | 0.056748 |
| Palatinose | 0.010973 | 3.97E-06 | 1.75E-07 | 0.005699 | 0.004136 | 0.004576 | 1.71E-07 | 1.74E-07 | 0.004064 |
| Melibiose | 0.060302 | 0.057582 | 0.054861 | 0.006805 | 0.000798 | 1.55E-07 | 0.017115 | 0.013526 | 0.011155 |
| Digalacturonic acid | 0.077102 | 0.063395 | 0.049688 | 1.81E-07 | 1.56E-07 | 0.050231 | 0.0232 | 0.021752 | 0.020304 |
| Isomaltose | 0.035982 | 0.023735 | 0.011489 | 0.01941 | 0.015135 | 0.016085 | 0.009979 | 0.010844 | 0.01171 |
| Loganin | 4.2E-07 | 0.100083 | 1.75E-07 | 0.003573 | 0.003536 | 0.003499 | 0.004202 | 0.00362 | 0.003039 |
| palatinitol | 0.067062 | 3.97E-06 | 1.75E-07 | 0.020494 | 0.026955 | 0.026915 | 0.030845 | 0.038287 | 0.045729 |
| Galactinol | 0.01421 | 0.189781 | 0.009254 | 0.008363 | 0.006536 | 0.003693 | 0.003902 | 0.003448 | 0.005903 |
| Cerotinic acid | 4.2E-07 | 0.315543 | 0.015212 | 0.015097 | 0.013761 | 0.014729 | 0.014199 | 0.015235 | 0.013162 |
| 21-Hydroxypregnenolone | 4.2E-07 | 3.97E-06 | 1.75E-07 | 0.000491 | 0.000414 | 0.000409 | 0.000409 | 1.74E-07 | 1.61E-07 |
| Tetrahydrocorticosterone | 4.2E-07 | 3.97E-06 | 1.75E-07 | 0.002433 | 1.56E-07 | 1.55E-07 | 0.002145 | 0.002201 | 0.002258 |
| 3,7,12-Trihydroxycoprostane | 0.024887 | 0.202805 | 0.008626 | 0.012283 | 0.008546 | 0.007703 | 0.008986 | 0.007922 | 0.010976 |
| Cholesterol | 4.2E-07 | 3.97E-06 | 1.75E-07 | 0.001076 | 0.0011 | 0.001125 | 1.71E-07 | 1.74E-07 | 1.61E-07 |
| Zymosterol | 0.176848 | 1.621833 | 0.89934 | 0.148426 | 0.115477 | 0.122627 | 0.125922 | 0.142718 | 0.091288 |
| 5-alpha-Cholestan | 0.0017 | 0.022015 | 1.75E-07 | 0.000604 | 0.000643 | 0.000759 | 0.00063 | 0.000819 | 0.000654 |
| Ergosterol | 0.768383 | 6.024179 | 1.75E-07 | 0.385229 | 0.26837 | 0.3268 | 0.325334 | 0.299365 | 0.257163 |
| Cholic acid | 4.2E-07 | 3.97E-06 | 0.001084 | 0.000957 | 0.000984 | 0.001012 | 0.001177 | 1.74E-07 | 1.61E-07 |
| Stigmasterol | 0.001271 | 0.012837 | 1.75E-07 | 0.000261 | 0.000307 | 0.000354 | 1.71E-07 | 1.74E-07 | 1.61E-07 |
| 24,25-Dihydrolanosterol | 0.910567 | 7.079317 | 1.75E-07 | 0.366073 | 0.271007 | 0.293856 | 0.341462 | 0.314499 | 0.287535 |
| Sitosterol | 0.023849 | 0.20057 | 1.75E-07 | 0.013546 | 0.008868 | 0.010266 | 0.011883 | 0.006785 | 0.008892 |
| Cholestane-3,5,6-triol | 1.410639 | 5.26399 | 0.256024 | 0.373391 | 0.418358 | 0.141361 | 0.432611 | 0.563462 | 0.28566 |

^1^The relative concentration of 316 indentified metabolites was the total mass of the signal integration area. Metabolites annotation was performed by importing the normalized data into the Simca software (version 14) to detect differentially expressed metabolites, and the NIST (http://www.nist.gov/index.html) and KEGG (http://www.genome.jp/kegg/) commercial databases were used to search for metabolites.
