# Supplementary material for: Modulation of Metabolome and Bacterial Community in Whole Crop Corn Silage by Inoculating Homofermentative Lactobacillus plantarum and Heterofermentative Lactobacillus buchneri
Source: Front Microbiol. 2019 Jan 23;9:3299. doi: 10.3389/fmicb.2018.03299 (PMC6352740; doi:10.3389/fmicb.2018.03299)
Supplement: TABLE S2 — The contribution of 316 indentified metabolites to the first principal component (PC1) and standard error. [file Table_2.DOCX]

**Modulation of metabolome and bacterial community in whole crop corn silage by inoculating homofermentative *Lactobacillus plantarum* and heterofermentative *Lactobacillus buchneri***

**Running title: Metabolome and microbiota in silage**

Dongmei Xu^1,2^, Wencan Ke^1,2^, Wurong Ding^1,2^, Fuhou Li^2,3^, Ping Zhang ^1,2^, Xusheng Guo^1,2^*****

^1^State Key Laboratory of Grassland and Agro-ecosystems, School of Life Sciences, Lanzhou University, Lanzhou 730000, PR China, guoxsh07@lzu.edu.cn

^2^Probiotics and Biological Feed Research Center, Lanzhou University, Lanzhou 730000, PR China

^3^Stay Key Laboratory of Grassland Agro-ecosystems, College of Pastoral Agriculture Science and Technology, Lanzhou University, Lanzhou 730020, China.

**TABLE S2** The contribution of 316 indentified metabolites to the first principal component (PC1) and standard error

| Peak | PC1 | SE |
| --- | --- | --- |
| Pyruvic acid | 0.000575388 | 0.000287397 |
| Lactic acid | 0.0330192 | 0.0365312 |
| Glycolic acid | 5.88E-05 | 4.22E-05 |
| Alanine | -3.74E-05 | 0.000270239 |
| 2-keto-isovaleric acid | 2.43E-05 | 1.09E-05 |
| Hydroxylamine | 0.00605446 | 0.017798 |
| 2-Hydroxybutanoic acid | -0.00322354 | 0.00700737 |
| Sarcosine | 0.00329784 | 0.00113388 |
| 3-Hydroxypropionic acid | -0.000215867 | 0.000161281 |
| 3-Hydroxybutyric acid | 0.0353409 | 0.0130762 |
| 3-Hydroxypyridine | 0.000832886 | 0.00135923 |
| Sulfuric acid | -0.00268689 | 0.00114228 |
| 4-Aminobutyric acid | 0.0157537 | 0.0151352 |
| Benzyl alcohol | -3.79E-06 | 3.63E-06 |
| N-Methyl-DL-alanine | 5.76E-05 | 9.71E-05 |
| 2-amino-2-methylpropane-1,3-diol | 0.00267861 | 0.00809597 |
| Lactamide | -0.000328704 | 0.000281093 |
| 2-Amino-3-methyl-1-butanol | 0.000364566 | 0.000799428 |
| O-methylthreonine | -0.0460155 | 0.0585157 |
| Succinate semialdehyde | 8.81E-05 | 6.55E-05 |
| Malonic acid | 0.000124158 | 0.000268481 |
| Norleucine | 0.000117495 | 5.75E-05 |
| Methylmalonic acid | 0.00208755 | 0.00238378 |
| alpha-Ketoisocaproic acid | 0.00186311 | 0.000277403 |
| Carnitine | 6.92E-07 | 4.97E-05 |
| Valine | -0.0325692 | 0.0252172 |
| Canavanine degr prod | 0.000183648 | 0.000203502 |
| Pyrophosphate | 5.97E-05 | 0.000166224 |
| 2-Butyne-1,4-diol | 6.35E-05 | 3.09E-05 |
| Guaiacol | -3.11E-05 | 6.14E-05 |
| 4-Hydroxybutyrate | 0.0010439 | 0.000474902 |
| 2-Ketoadipate | 0.000369387 | 0.00011363 |
| Dihydroxyacetone | -6.78E-05 | 9.82E-05 |
| Benzoic acid | 0.000548965 | 0.000215586 |
| Oxamic acid | -0.00127157 | 0.000825404 |
| Ethanolamine | -0.0540437 | 0.0392533 |
| Glutaraldehyde | -4.19E-05 | 0.000140433 |
| Leucine | 0.0255728 | 0.00861852 |
| 2-Deoxyerythritol | 0.0142285 | 0.00550374 |
| N-Cyclohexylformamide | 0.000499079 | 0.000151257 |
| 4-Vinylphenol | -4.29E-05 | 2.32E-05 |
| Isoleucine | -0.0197443 | 0.0173048 |
| Proline | 0.0728206 | 0.0356498 |
| Phenylacetic acid | 0.000306673 | 0.000100014 |
| Glycine | -0.000137782 | 8.87E-05 |
| 1,3-Cyclohexanedione | 3.33E-06 | 5.87E-06 |
| Succinic acid | 0.0573829 | 0.0257215 |
| Catechol | 1.20E-05 | 0.000763986 |
| 2,2-Dimethylsuccinic acid | -4.74E-05 | 4.83E-05 |
| 4-Acetylbutyric acid | 0.00316341 | 0.000881053 |
| D-Glyceric acid | -0.0011903 | 0.000877549 |
| Uracil | -0.0193382 | 0.013395 |
| 2,3-Dimethylsuccinic acid | -1.50E-05 | 1.32E-05 |
| Fumaric acid | 0.000232586 | 0.000141529 |
| Serine | 0.124406 | 0.068875 |
| 3-Hydroxynorvaline | -0.00149552 | 0.00124689 |
| Cycloleucine | 0.000645138 | 0.000904823 |
| 3-Cyanoalanine | 0.000838525 | 0.000444687 |
| 3-Methylamino-1,2-propanediol | 0.00109103 | 0.000496875 |
| L-Allothreonine | 0.00837663 | 0.00601707 |
| 4-Methyl-5-thiazolethanol | -0.000159 | 0.0001751 |
| 4-Methylcatechol | -1.30E-05 | 1.07E-05 |
| O-Acetylserine | -5.49E-05 | 0.000143131 |
| Glutaric acid | -1.40E-05 | 3.05E-05 |
| Thymine | -0.00602327 | 0.00446884 |
| DL-Anabasine | -0.000357195 | 0.000317417 |
| Biphenyl | -5.33E-05 | 3.98E-05 |
| 2-Methylglutaric acid | 0.000292065 | 0.000160226 |
| Methyl trans-cinnamate | -2.69E-05 | 0.000559886 |
| N-Ethylglycine | -0.00011663 | 0.00034607 |
| Aspartic acid | 0.000113173 | 7.05E-05 |
| beta-Alanine | -0.00373002 | 0.00466389 |
| D-Erythronolactone | -5.37E-05 | 0.000124444 |
| Salicyl alcohol | 6.58E-05 | 9.76E-06 |
| L-Homoserine | -0.00141331 | 0.00110539 |
| Erythrose | 0.000334964 | 0.000197159 |
| L-Threose | 0.0228372 | 0.0461288 |
| Capric acid | -1.90E-05 | 4.54E-05 |
| 3-Aminoisobutyric acid | 0.00067717 | 0.000758788 |
| 2-Hydroxyacetophenone | 6.17E-05 | 0.000163546 |
| Aminomalonic acid | -0.00179082 | 0.00118101 |
| (S)-Mandelic acid | 0.000174643 | 0.000326449 |
| Bis(2-hydroxypropyl)amine | 0.000101166 | 5.36E-05 |
| N-Ethylmaleamic acid | 0.000143674 | 8.80E-05 |
| L-Malic acid | 6.93E-05 | 9.62E-05 |
| 4-Hydroxycyclohexanecarboxylic acid | -5.19E-05 | 0.000117214 |
| 5,6-Dihydrouracil | 0.00156085 | 0.0013483 |
| Ethyl cinnamate | -4.25E-05 | 0.000100825 |
| Threitol | 0.000887868 | 0.00253524 |
| 1,5-Anhydroglucitol | 0.000602348 | 0.000286482 |
| N-Acetyl-L-leucine | -0.000458884 | 0.000465252 |
| 4-Acetamidobutyric acid | -0.00235735 | 0.00175273 |
| Methionine | 0.000214048 | 0.000310109 |
| Iminodiacetic acid | 0.0540059 | 0.104232 |
| Oxoproline | 0.0890827 | 0.187057 |
| Cytosin | -0.00250364 | 0.0022844 |
| Benzoylformic acid | -1.27E-05 | 2.74E-05 |
| 4-Aminobutyric acid | 0.0019852 | 0.000146633 |
| 4-Hydroxyquinazoline | -0.000107403 | 0.000192105 |
| Maleamate | -0.000456388 | 0.000385419 |
| Pyrogallol | -0.000130479 | 0.000101006 |
| Nornicotine | -0.00032118 | 0.000290038 |
| Malonamide | -0.000494466 | 0.000504596 |
| Threonic acid | 0.00128155 | 0.0013972 |
| Creatine | -0.0003412 | 0.000459097 |
| Phenylethylamine | 0.00146971 | 0.00148104 |
| 2-Hydroxy-3-isopropylbutanedioic acid | -0.00368387 | 0.0044058 |
| (2R,3S)-2-Hydroxy-3-isopropylbutanedioic acid | -0.000394146 | 0.000262903 |
| threo-beta-Hyrdoxyaspartate | 0.00380639 | 0.00134948 |
| 3-Phenyllactic acid | -0.014634 | 0.00950747 |
| Phosphoglycolic acid | 4.91E-05 | 4.62E-05 |
| 3-Hydroxy-3-methylglutaric acid | -0.00123394 | 0.00092611 |
| Hexadecane | -0.000917203 | 0.000739003 |
| Digitoxose | 0.0513171 | 0.0202956 |
| 3-Hydroxyphenylacetic acid | -1.15E-05 | 1.41E-05 |
| Glutamic acid | -0.00849078 | 0.00971684 |
| Phenylalanine | -0.0536908 | 0.0333382 |
| 4-Hydroxybenzoic acid | -0.00312353 | 0.00216689 |
| Creatine degr | -0.000903407 | 0.000674498 |
| Tartaric acid | -0.000169412 | 0.000171731 |
| 5-Aminovaleric acid | -0.00105293 | 0.00079277 |
| 4-Hydroxy-3-methoxybenzyl alcohol | -8.20E-06 | 2.26E-05 |
| 4-Hydroxyphenylacetic acid | 0.000852786 | 0.00104115 |
| Lyxose | 0.201429 | 0.0888084 |
| 1,3-Diaminopropane | -0.00590467 | 0.00575772 |
| Allose | -8.62E-05 | 0.000175459 |
| Lauric acid | -0.000521808 | 0.00049162 |
| Xylose | 0.00651995 | 0.00272971 |
| Asparagine | 0.000417965 | 0.00070783 |
| Ribonic acid, gamma-lactone | 0.00524994 | 0.0051257 |
| Ribose | -0.000184038 | 0.000663026 |
| 3-Ureidopropionate | -0.000256276 | 0.00023204 |
| Xylitol | 0.0198697 | 0.00723065 |
| Biuret | -0.00174306 | 0.00289588 |
| 6-deoxy-D-Glucose | -0.000524485 | 0.00109815 |
| Fucose | 0.00414484 | 0.00801303 |
| Ribitol | -0.00108555 | 0.000850818 |
| Putrescine | -0.000134359 | 0.000429104 |
| Glutaconic acid | -1.19E-05 | 0.00582744 |
| Lyxonic acid, 1,4-lactone | -2.60E-05 | 8.89E-05 |
| Orotic acid | 0.000257015 | 0.000134697 |
| Carbobenzyloxy-L-leucine degr3 | 7.92E-05 | 0.00108059 |
| Hexachlorobenzene | 0.00111449 | 0.00589107 |
| flavin adenine degrad product | -0.000219428 | 0.000162528 |
| Aconitic acid | -9.49E-05 | 0.000719408 |
| Diglycerol | 0.00177696 | 0.000398691 |
| D-(glycerol 1-phosphate) | 8.81E-05 | 2.98E-05 |
| Glucose-1-phosphate | -0.00349925 | 0.00238119 |
| 3,6-Anhydro-D-galactose | 0.000151534 | 0.00022377 |
| 5,6-Dimethylbenzimidazole | -0.0222648 | 0.00759668 |
| 3-(4-hydroxyphenyl)Propionic acid | -0.0643249 | 0.0131545 |
| 4-Hydroxy-3-methoxybenzoic acid | -0.00271842 | 0.00176113 |
| Gentisic acid | -0.00170124 | 0.00118712 |
| N-Acetyl-L-glutamic acid | 0.000501088 | 0.00190351 |
| Methionine sulfoxide | 6.50E-05 | 0.000614155 |
| 5-Aminoimidazole-4-carboxamide | -0.000648088 | 0.000393128 |
| 2-deoxy-D-Glucose | 0.000128238 | 4.28E-05 |
| 2-Deoxy-D-Galactose | -0.0114388 | 0.00484998 |
| Methoxamedrine | -0.000131134 | 9.38E-05 |
| Azelaic acid | -0.000396954 | 0.000209751 |
| Cysteinylglycine | -0.000264031 | 0.0011263 |
| Shikimic acid | -6.48E-06 | 9.80E-06 |
| Hypoxanthine | -0.00112016 | 0.00161162 |
| Ornithin | -0.0591547 | 0.0420487 |
| alpha-D-glucosamine 1-phosphate | -0.00219195 | 0.00465106 |
| Isocitric acid | -0.00196695 | 0.00251737 |
| 3,4-Dihydroxybenzoic acid | 0.00854463 | 0.00411408 |
| N-Acetylisatin | -0.000321071 | 0.00038674 |
| Synephrine | -0.000161124 | 0.000329236 |
| Myristic acid | 0.000647823 | 0.000254489 |
| Guanidinosuccinic acid | -0.000265294 | 0.000138429 |
| Quinic acid | 0.000233737 | 0.000166632 |
| Tagatose | -0.00610073 | 0.00881097 |
| Allo-inositol | -8.41E-05 | 0.00017515 |
| Sorbose | -4.28E-05 | 3.06E-05 |
| Fructose | 0.000674705 | 0.000228159 |
| Adenine | -0.0122193 | 0.00996427 |
| Adipamide | 0.00117008 | 0.00401908 |
| Vanillylmandelic acid | -0.00437915 | 0.00355967 |
| Gluconic lactone | -0.000104354 | 8.75E-05 |
| Mannose | -0.000428147 | 0.000966473 |
| D-Altrose | 0.00170646 | 0.000627291 |
| 2-Keto-L-Gulonic acid | -0.000324198 | 0.000682479 |
| Glucose | 2.98E-05 | 0.000181992 |
| D-Talose | -0.0143609 | 0.0166133 |
| dl-p-Hydroxyphenyllactic acid | 0.00200273 | 0.00348117 |
| Galactose | -0.0031244 | 0.038693 |
| Methyl Palmitoleate | 0.00262733 | 0.0011094 |
| Tyramine | -0.121024 | 0.0859232 |
| Lysine | -0.0122881 | 0.00704621 |
| Mannitol | -0.00139616 | 0.00110657 |
| Sorbitol | -8.07E-05 | 9.53E-05 |
| D-Galacturonic acid | 0.000425161 | 0.00474961 |
| Tyrosine | -0.0039995 | 0.00172795 |
| Coniferyl alcohol | -0.000116885 | 0.000130629 |
| 4-Hydroxycinnamic acid | -0.0069485 | 0.00520942 |
| Pentadecanoic acid | -0.000102972 | 6.18E-05 |
| Conduritol-β-epoxide | -0.000263016 | 0.000193651 |
| Gallic acid | -9.20E-05 | 0.000132998 |
| Sinapyl alcohol | 0.00034449 | 8.86E-05 |
| 4-Hydroxy-3-methoxycinnamaldehyde | -0.000235918 | 0.000254926 |
| 3,5-Dihydroxyphenylglycine | -0.000116964 | 0.00023739 |
| indole-3-Acetic acid | -0.000235111 | 0.000504874 |
| Canavanine | -1.83E-05 | 3.16E-05 |
| 2,6-Diaminopimelic acid | -9.19E-05 | 9.13E-05 |
| Lipoic acid | -2.28E-05 | 2.36E-05 |
| Gly-pro | 6.34E-05 | 6.59E-05 |
| N-alpha-Acetyl-L-ornithine | 0.000240323 | 0.00130963 |
| Galactonic acid | 0.000178287 | 0.000114921 |
| Gluconic acid | 0.000509554 | 0.000718335 |
| Saccharic acid | -0.000548489 | 0.00367146 |
| Xanthine | 0.00261115 | 0.00665641 |
| Glucosaminic acid | -0.000783001 | 0.000695967 |
| Palmitoleic acid | 7.92E-05 | 4.84E-05 |
| 4-Hydroxymethyl-3-methoxyphenoxyacetic acid | -0.00123858 | 0.000603744 |
| N-Carbamylglutamate | 0.000243174 | 0.00156761 |
| Mucic acid | 0.00149104 | 0.0007633 |
| Palmitic acid | 0.119209 | 0.047543 |
| Isopropyl-beta-D-thiogalactopyranoside | -3.81E-05 | 7.38E-05 |
| N,N-Dimethylarginine | -0.000187475 | 0.000436731 |
| N-Acetyl-D-galactosamine | 0.000683325 | 0.00105255 |
| myo-Inositol | 0.00331198 | 0.0108766 |
| Linoleic acid methyl ester | 0.0137426 | 0.0144427 |
| Ferulic acid | 0.0248886 | 0.00404126 |
| N-Acetyl-beta-D-mannosamine | 0.0174187 | 0.00328081 |
| indole-3-Acetamide | 0.000956318 | 0.000227388 |
| d-Glucoheptose | 0.000432641 | 0.000215415 |
| trans-3,5-Dimethoxy-4-hydroxycinnamaldehyde | -5.09E-05 | 3.23E-05 |
| Guanine | 0.00184609 | 0.00284506 |
| Glucoheptonic acid | 0.0040995 | 0.0020783 |
| Flavanone | -0.000120234 | 8.86E-05 |
| 3,4-Dihydroxycinnamic acid | -0.000608977 | 0.000282107 |
| Caffeic acid | -5.35E-05 | 4.11E-05 |
| cis-Phytol | -0.00010111 | 6.92E-05 |
| Heptadecanoic acid | 0.000932944 | 0.000341785 |
| 6-Hydroxy caproic acid dimer | 5.15E-05 | 4.43E-05 |
| Indolelactate | 0.000130274 | 0.000193981 |
| Glutathione | 0.0230768 | 0.0109797 |
| Phytol | 0.0146325 | 0.00772064 |
| L-Kynurenine | -0.000198008 | 0.000353021 |
| 3-Hydroxypalmitic acid | -0.00210991 | 0.00132879 |
| beta-Mannosylglycerate | 0.0107044 | 0.0052091 |
| Linoleic acid | 0.0042213 | 0.00138868 |
| Oleic acid | -0.00118557 | 0.000617069 |
| Linolenic acid | -0.00795721 | 0.00889577 |
| 2-Aminoethanethiol | 0.0501002 | 0.0393696 |
| trans-Sinapinic acid | 0.00616705 | 0.00147744 |
| Stearic acid | 0.0340202 | 0.0185166 |
| Pyridoxal phosphate | 0.00021548 | 0.000257382 |
| Xanthurenic acid | -0.000109671 | 7.34E-05 |
| spermidine | 6.29E-05 | 5.22E-05 |
| alpha-Santonin | -2.67E-06 | 0.000177646 |
| Fructose-6-phosphate | 1.82E-05 | 5.52E-05 |
| Atropine | 0.00136749 | 0.000611966 |
| Phenyl beta-D-glucopyranoside | -8.26E-05 | 0.00015144 |
| Glucose-6-phosphate | 2.38E-05 | 0.000234829 |
| 3-Hydroxyflavone | -0.000179144 | 4.35E-05 |
| Purine riboside | 0.000278581 | 0.000215434 |
| Arachidonic acid | 0.000217658 | 8.31E-05 |
| 1-Methyladenosine | -0.000421882 | 0.00124287 |
| Abietic acid | 1.38E-05 | 0.000261012 |
| cis-Gondoic acid | -5.88E-05 | 8.32E-05 |
| 6-Phosphogluconic acid | 0.00539385 | 0.00168742 |
| Arachidic acid | 0.00316265 | 0.000931862 |
| D-erythro-sphingosine | -3.36E-05 | 3.41E-05 |
| 4-Vinylphenol dimer | -1.69E-05 | 5.36E-05 |
| Uridine | 0.000183021 | 9.41E-05 |
| 5-Methoxytryptamine | -3.36E-05 | 0.000534426 |
| Neohesperidin | 0.000762877 | 0.000198696 |
| Salicin | -0.000266915 | 0.000149883 |
| Homocystine | 0.000296678 | 0.00132016 |
| 2-Monopalmitin | 8.75E-05 | 0.000236154 |
| Arbutin | 0.000134011 | 8.67E-05 |
| Androsterone | -0.000278804 | 0.000253899 |
| kyotorphin | 0.00142832 | 0.00035989 |
| 1-Monopalmitin | 0.00130516 | 0.000702717 |
| Phytosphingosine | 8.44E-05 | 3.36E-05 |
| Prostaglandin | 0.000196084 | 7.03E-05 |
| Sucrose | 0.000344468 | 0.000366938 |
| Behenic acid | 0.00153499 | 0.000456454 |
| Androstanediol | 4.79E-06 | 1.90E-05 |
| Lactose | 0.00633802 | 0.00379755 |
| Dihydrotestosterone | 2.69E-06 | 3.39E-05 |
| Lactulose | 2.73E-05 | 6.62E-05 |
| 11-beta-prostaglandin-F-2-alpha | -0.000509256 | 0.00128463 |
| Cellobiose | -0.00080029 | 0.000671707 |
| Trehalose | 0.00126481 | 0.00209338 |
| Monoolein | 3.04E-06 | 8.16E-06 |
| Sophorose | -0.000194242 | 0.000254194 |
| Leucrose | -2.07E-05 | 0.00028846 |
| Monostearin | 0.000490647 | 0.000207553 |
| Gentiobiose | -2.10E-05 | 5.91E-05 |
| Prostaglandin | 2.19E-05 | 1.28E-05 |
| Squalene | -0.00110628 | 0.000445798 |
| prunin degr. Prod. | -0.000115981 | 0.00108799 |
| Lignoceric acid | -0.00103659 | 0.000717188 |
| Palatinose | 0.000145507 | 0.000177857 |
| Melibiose | 0.00108176 | 0.000768418 |
| Digalacturonic acid | 0.00112966 | 0.000353696 |
| Isomaltose | 0.000417406 | 0.000224421 |
| Loganin | -8.00E-05 | 6.39E-05 |
| palatinitol | 0.000420338 | 0.00130399 |
| Galactinol | 0.000186878 | 2.42E-05 |
| Cerotinic acid | -0.000247073 | 0.000204304 |
| 21-Hydroxypregnenolone | -5.01E-06 | 8.87E-06 |
| Tetrahydrocorticosterone | -3.64E-05 | 2.75E-05 |
| 3,7,12-Trihydroxycoprostane | 0.000277448 | 0.000194593 |
| Cholesterol | -6.94E-06 | 1.45E-05 |
| Zymosterol | 0.00445799 | 0.021294 |
| 5-alpha-Cholestan | 1.48E-05 | 2.49E-05 |
| Ergosterol | 0.00705024 | 0.0119103 |
| Cholic acid | -8.27E-06 | 1.42E-05 |
| Stigmasterol | 2.05E-05 | 1.37E-05 |
| 24,25-Dihydrolanosterol | 0.00932092 | 0.0137888 |
| Sitosterol | 0.000212739 | 0.00044931 |
| Cholestane-3,5,6-triol | 0.0174151 | 0.0100716 |
